# Supplementary material for: Molecular architecture of OXGR1 reveals an evolutionary conserved mechanisms for metabolite surveillance
Source: EMBO J. 2026 Jun 3;45(14):4931–55. doi: 10.1038/s44318-026-00823-y (PMC13372822; doi:10.1038/s44318-026-00823-y)
Supplement: Supplementary file 1 — Appendix [file 44318_2026_823_MOESM1_ESM.pdf]

Appendix

Molecular architecture of OXGR1 reveals an evolutionary conserved mechanisms for metabolite surveillance

Xinyue Zhang[1,2]*, Yujie Lu[1,2]*, Xinheng He[2,3,4]*, Shimeng Guo[2]*, Changyao Li[2,4,5], Yu Wang[2,3,6], Yuan Gao[2,3], Juxia Yao[2,3], Qingning Yuan[2,7,8], Yinshan Tang[2,3], Jing Hu[2,3], Wen Hu[2,8], Zijuan Luo[2,3], Kai Wu[2,8], Yue Wang[2], Wanchao Yin[2,3,9], Xin Xie[1,2,3,6]✉, H. Eric Xu[1,2,3,5,7]✉, Heng Liu[2]✉

[1]School of Chinese Materia Medica, Nanjing University of Chinese Medicine, Nanjing 210023. China

[2]State Key Laboratory of Drug Research, Shanghai Institute of Materia Medica, Chinese Academy of Sciences, Shanghai 201203, China

[3]University of Chinese Academy of Sciences, Beijing 100049, China

[4] Lingang Laboratory, Shanghai, 200031, China

[5] School of Life Science and Technology, ShanghaiTech University, 201210 Shanghai, China.

[6]School of Pharmaceutical Science and Technology, Hangzhou Institute for Advanced Study, University of Chinese Academy of Sciences, Hangzhou 310024, China

[7]Research Center for Medicinal Structural Biology, National Research Center for Translational Medicine at Shanghai, State Key Laboratory of Medical Genomics, Ruijin Hospital affiliated to Shanghai Jiao Tong University School of Medicine, Shanghai 200025, Ch ina

[8]The Shanghai Advanced Electron Microscope Center, Shanghai Institute of Materia Medica, Chinese Academy of Sciences, Shanghai, China

[9]Zhongshan Institute for Drug Discovery, Shanghai Institute of Materia Medica, Chinese Academy of Sciences, Guangdong 528400, China

*These authors contributed equally.

✉Correspondence: xxie@simm.ac.cn (X.X.); eric.xu@simm.ac.cn (H.E.X.); and liuheng@simm.ac.cn (H.L.)

Table of Contents:

Appendix Figure S1	Page 3-4
Appendix Figure S2	Page 5-6
Appendix Figure S3	Page 7-8
Appendix Figure S4	Page 9
Appendix Figure S5	Page 10
Appendix Figure S6	Page 11
Appendix Figure S7	Page 12
Appendix Figure S8	Page 13
Appendix Figure S9	Page 14
Appendix Table S1	Page 15
Appendix Table S2	Page 16
Appendix Table S3	Page 17
Appendix Table S4	Page 18-19
Appendix Table S5	Page 20
Appendix Table S6	Page 21

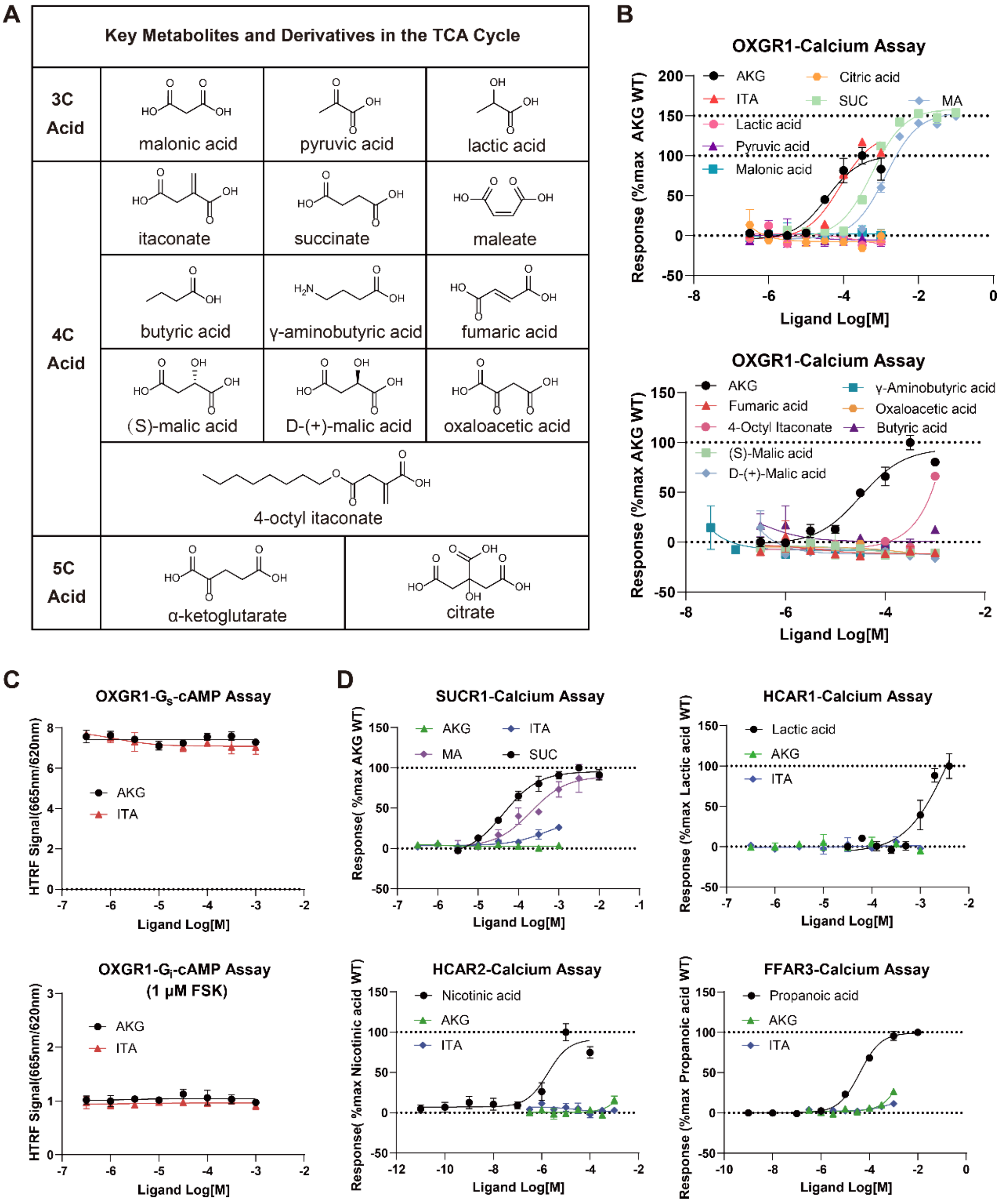

Appendix Figure S1. Functional characterization of TCA metabolites and derivatives in the activation of OXGR1.

(A) Chemical structures of investigated metabolites and derivatives;

(B-D) Concentration-response curves of the 15 metabolites and derivatives in activating OXGR1 through the calcium assay **(B)**, assessment of AKG and ITA activating OXGR1 through G_i/G_s pathway in cAMP assay **(C)**, and Concentration-response curves of the ITA and AKG in activating SUCR1, HCAR1/HCAR2, FFAR3 **(D)**. Data represent the mean ± S.E.M. from three independent experiments (*n* = 3), with three technical replicates performed for each

experiment.

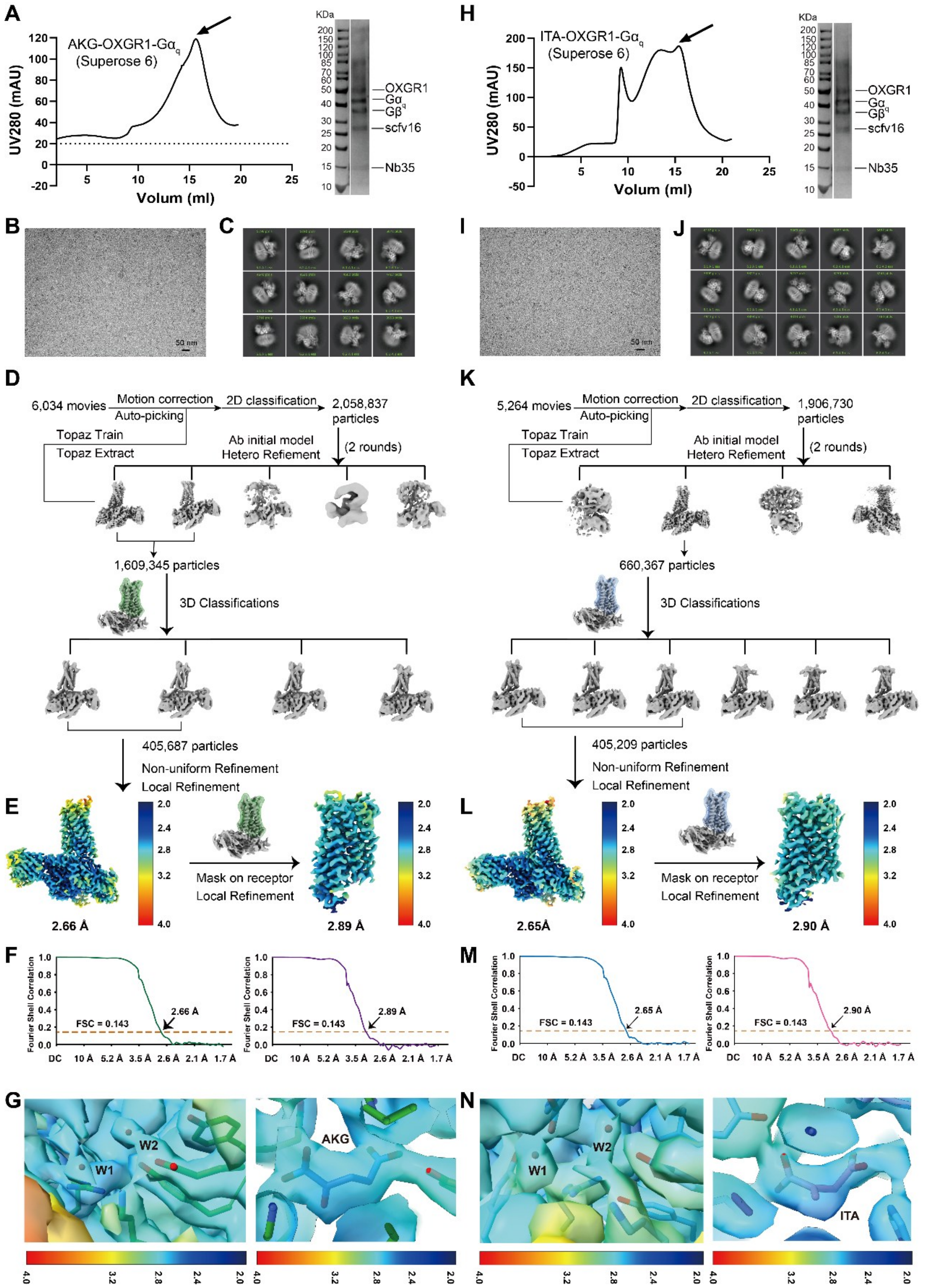

Appendix Figure S2. Purification and data-procession of AKG-OXGR1-G$_q$ and ITA-OXGR1-G$_q$ complex.

(A, H) Representative size exclusion chromatography (SEC) profiles and SDS-PAGE analysis of OXGR1-G$_q$ complex activated by AKG **(A)** and ITA **(H)**. Experiment was repeated at least

three times with similar results.

(B-C and I-J) Representative cryo-EM image and 2D classification averages of AKG-OXGR1-G_q complex

(D and K) Cryo-EM data processing flowcharts of AKG-OXGR1-G_q **(D)** and ITA-OXGR1-G_q complexes **(K)**.

(E and l) The global and local receptors density maps of AKG-OXGR1-G_q **(E)** and ITA-OXGR1-G_q **(l)** colored by local resolutions maps to a range of 2-4 Å.

(F and M) The Fourier shell correlation (FSC) curves of AKG-OXGR1-G_q, AKG-OXGR1 **(F)** and ITA-OXGR1-G_q, ITA -OXGR1 **(M)**. The global resolution of the final processed density map estimated at the FSC = 0.143 is 2.66 Å and 2.65 Å. The local resolution of the final processed receptor density map estimated at the FSC = 0.143 is 2.89 Å and 2.90 Å.

(G and N) The density of water molecules (W1/W2) and AKG **(G)**/ITA **(N)** in the in the OXGR1 binding pocket, colored by local resolution maps to a range of 2-4 Å, are shown at a contour level of 0.3.

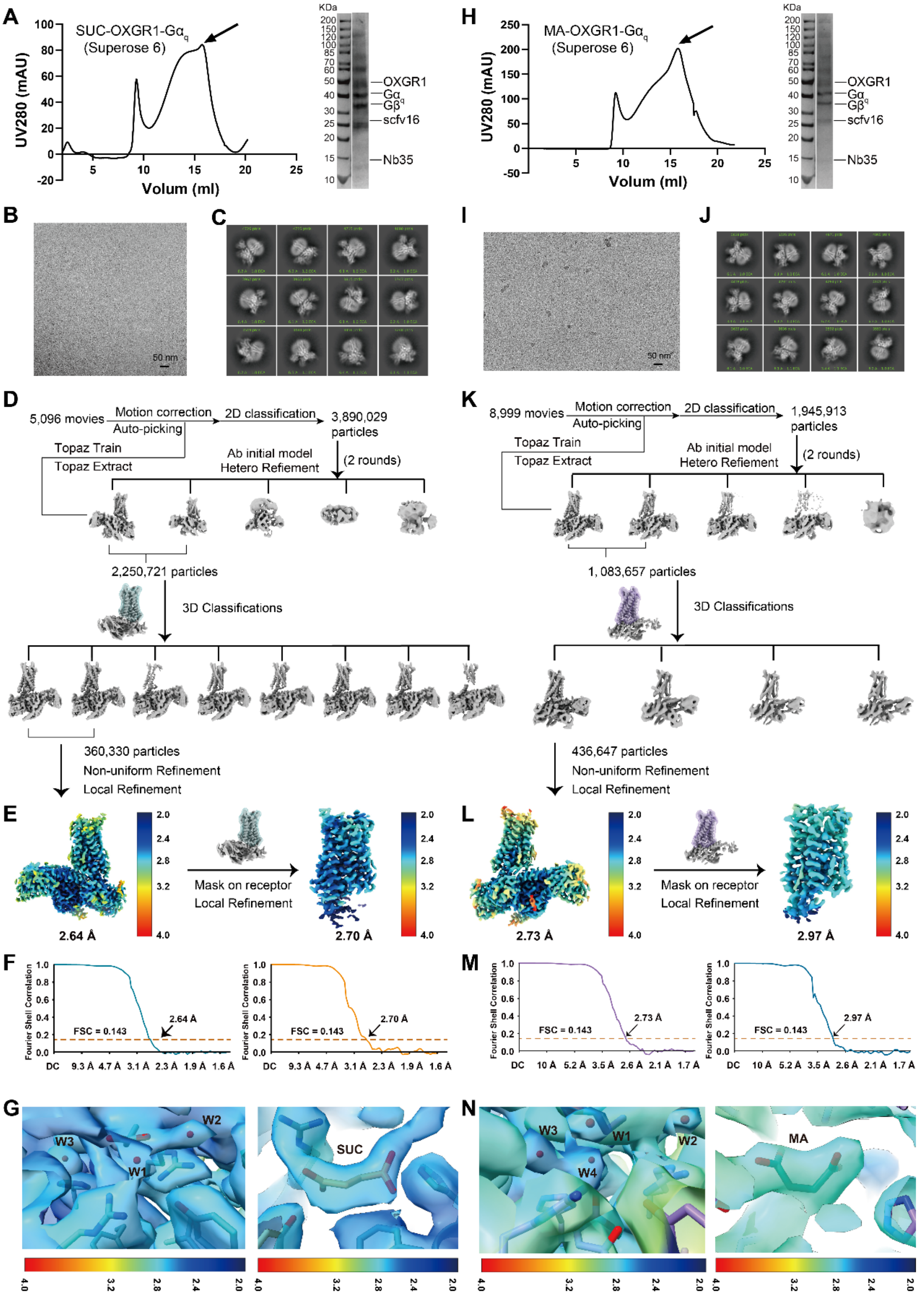

Appendix Figure S3. Purification and data-procession of SUC-OXGR1-G$_q$ and MA-OXGR1-G$_q$ complex.

(A and H) Representative size exclusion chromatography (SEC) profiles and SDS-PAGE analysis of OXGR1-G$_q$ complex activated by SUC **(A)** and MA **(H)**. Experiment was repeated

at least three times with similar results.

(B and I) Representative cryo-EM image of SUC-OXGR1-G_q **(B)** and MA-OXGR1-G_q complex **(I)**.

(C and J) Representative 2D classification averages of SUC-OXGR1-G_q **(C)** and MA-OXGR1-G_q complex **(J)**.

(D and K) Cryo-EM data processing flowcharts of SUC-OXGR1-G_q **(D)** and MA-OXGR1-G_q complexes **(K)**.

(E and l) The global density map of SUC-OXGR1-G_q **(E)** and MA-OXGR1-G_q **(l)** colored by local resolutions maps to a range of 2-4 Å.

(F and M) The Fourier shell correlation (FSC) curves of SUC-OXGR1-G_q **(F)** and MA-OXGR1-G_q **(M)**. The global resolution of the final processed density map estimated at the FSC = 0.143 is 2.64 Å and 2.73 Å. The local resolution of the final processed receptor density map estimated at the FSC = 0.143 is 2.70 Å and 2.97 Å.

(G and N) The density of water molecules (W1/W2/W3/W4) and SUC **(G)**/MA **(N)** in the in the OXGR1 binding pocket, colored by local resolution maps to a range of 2-4 Å, are shown at a contour level of 0.3.

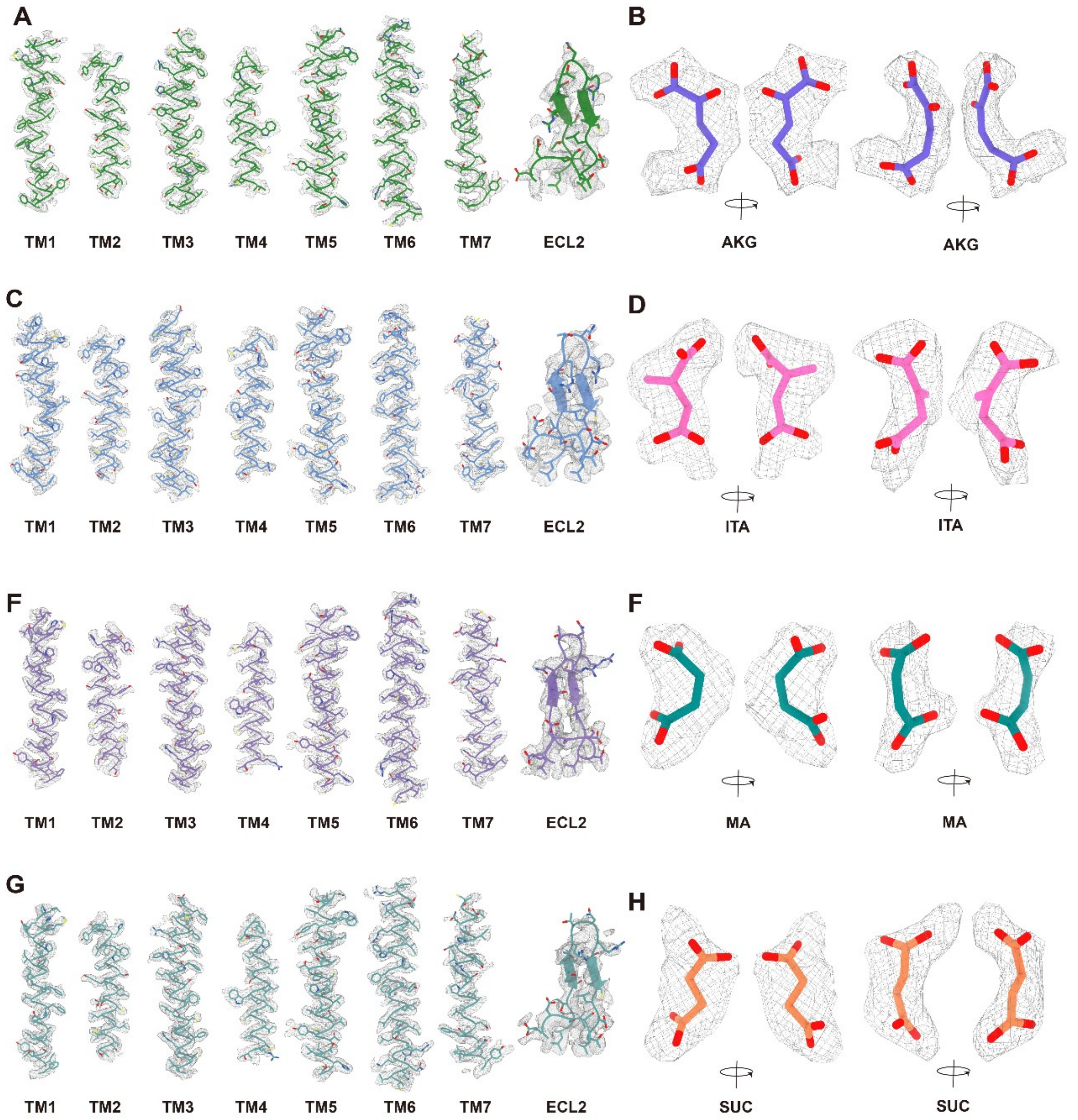

Appendix Figure S4. The density maps of protein and four ligands in OXGR1-G_q complex.

(A-H) The density maps of helices TM1-TM7 of transmembrane domain, extracellular loop ECL2 of OXGR1 **(A, C, E, G)** in OXGR1-G_q complex in the presence of AKG **(B)**/ITA**(D)** /SUC**(F)** /MA**(H)**. All protein density maps are shown at contour level of 0.5. The densities of respective ligands have been extracted from their local structures of receptors and shown in mesh presentation. All ligand density maps are shown at contour level of 0.3, and the local resolutions of AKG/ITA/SUC/MA-bound OXGR1 are 2.89Å, 2.90 Å, 2.70 Å, 2.97 Å, respectively.

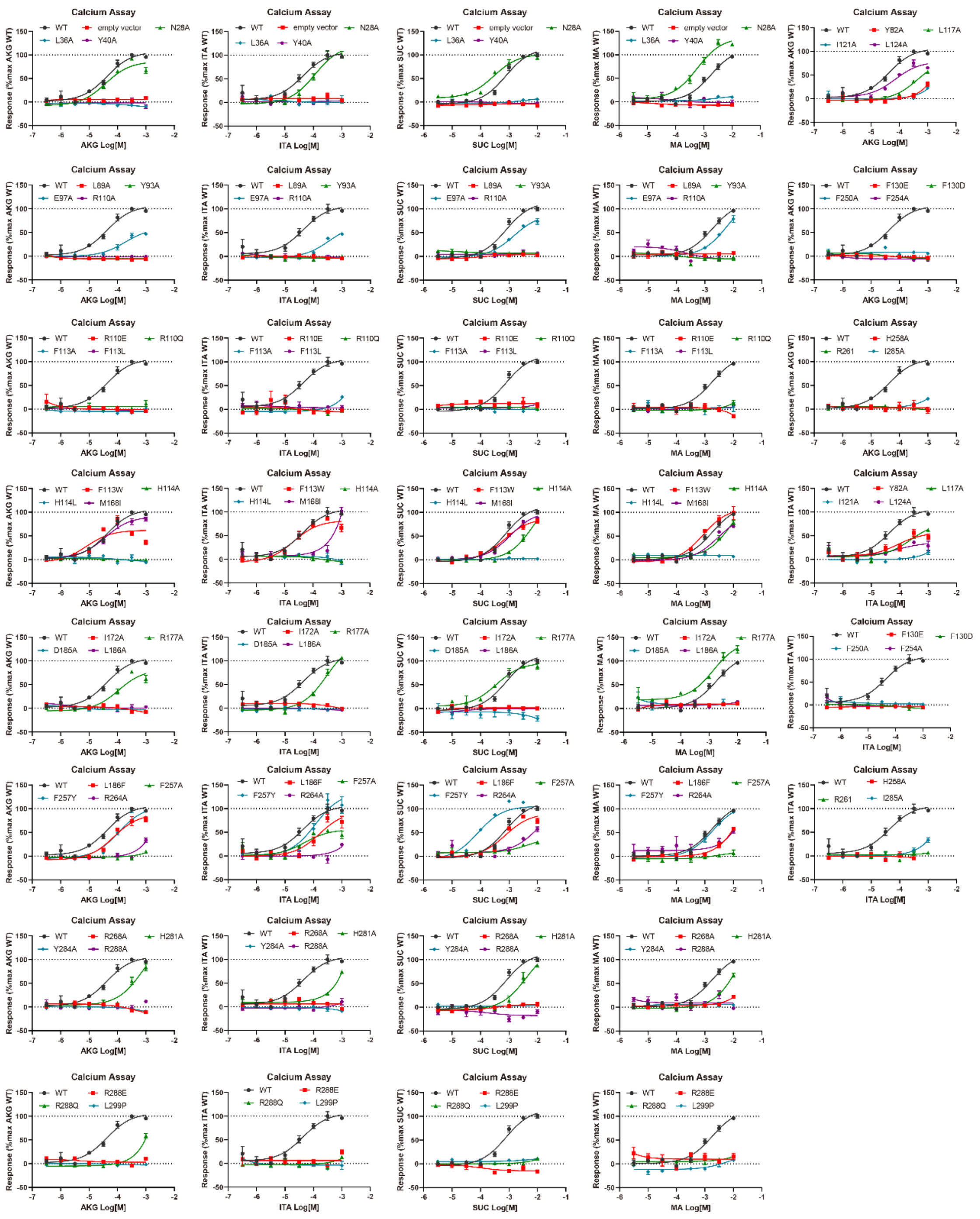

Appendix Figure S5. Mutation and functional assays indicate ligand effects on the activation of mutated OXGR1 variants.

Concentration-response curves show ligand-induced activation of mutated OXGR1 variants corresponding to ligand binding. Data represent the mean ± S.E.M. from three independent experiments ($n = 3$), with three technical replicates performed for each experiment. Three independent measurements refer to three different transfections.

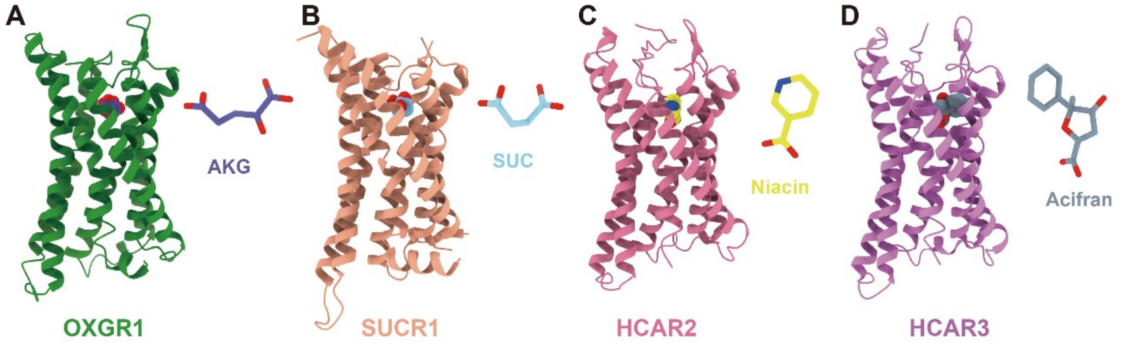

Appendix Figure S6. Different recognition modes of ligands in carboxylic acid-sensing receptors.

Binding pose of ligands in cryo-EM structures of OXGR1-AKG (**A**), SUC-SUCR1 **(B)** **(**PDB:8YKW), Niacin-HCA2 **(C)** (PDB:8K5B), and Acifran-HCAR3 **(D)** (PDB:8IHK). OXGR1 and SUCR1 share a horizontal ligand-binding mode (**A** and **B**), in contrast to the vertical binding pose adopted by HCAR2 and HCAR3(**C** and **D**).

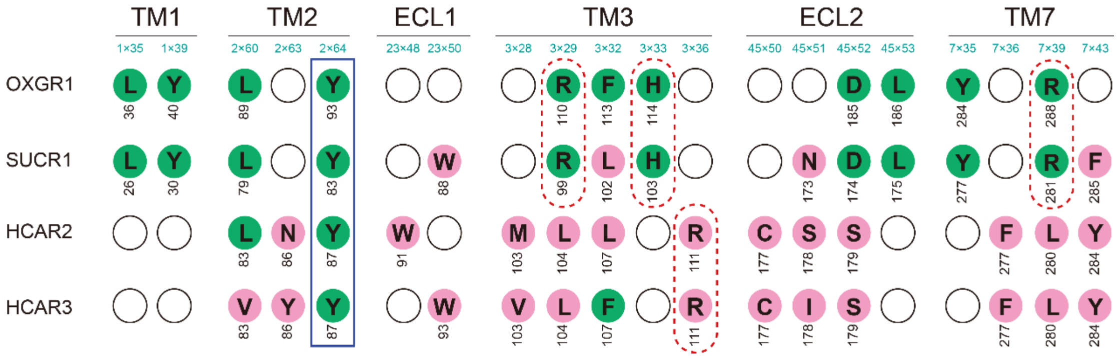

Appendix Figure S7. Key basic residues in OXGR1 binding pocket underlies selective dicarboxylate recognition.

Sequence alignment of OXGR1, SUCR1, HCAR2, and HCAR3 binding residues reveals a basic residue-rich environment in OXGR1. This feature is shared with SUCR1 but absent in HCAR2/3, which is consistent with ligand orientation in Figure. 5A-C. Identical residues to the OXGR1 binding pocket are highlighted with green backgrounds; divergent residues with pink. Fully conserved residues across the receptor family are blue-boxed; non-conserved basic residues are red-dashed-circled.

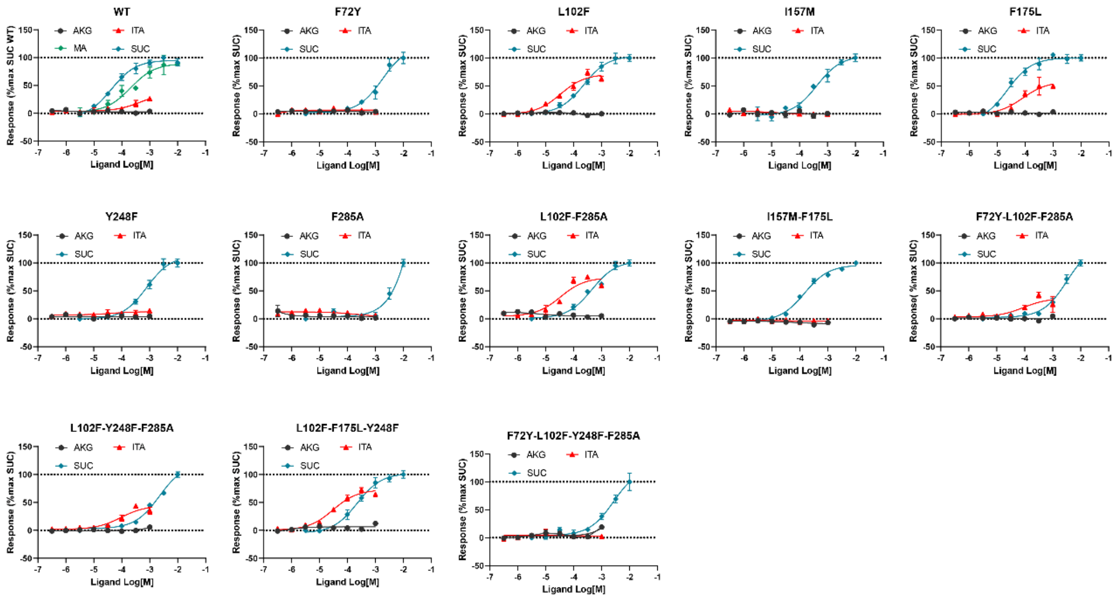

Appendix Figure S8. Mutation and functional assays indicate ligand effects on the activation of mutated SUCR1 variants.

Concentration-response curves show ligand-induced activation of mutated SUCR1 variants corresponding to ligand binding. Data represent the mean ± S.E.M. from three independent experiments ($n = 3$), with three technical replicates performed for each experiment. Three independent measurements refer to three different transfections.

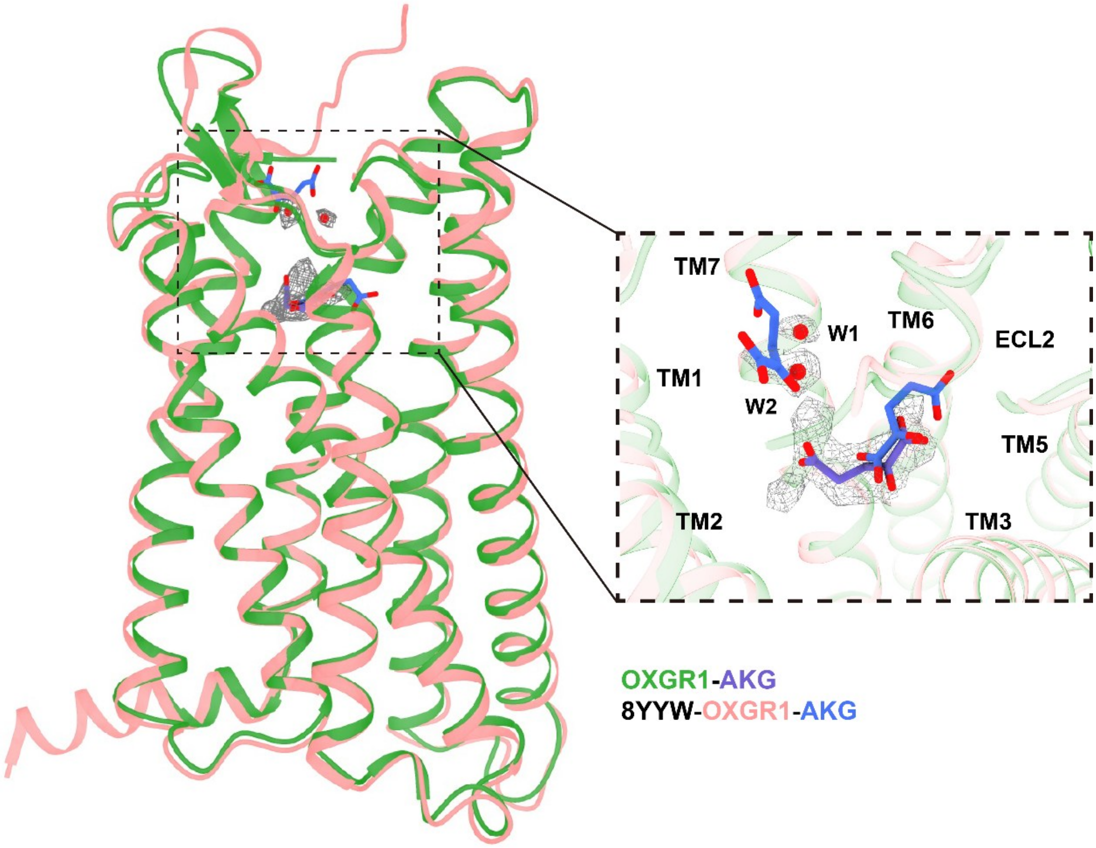

Appendix Figure S9. Comparison of ligand recognition between OXGR1-AKG complex and recently released OXGR1-AKG structure (PDB ID: 8YYW).

The AKG of our cryo-EM structure overlaid on corresponding density map in gray mesh shown at contour level of 0.3, with AKG in the orthosteric pocket. Superposition with 8YYW showing partial overlap of primary AKG. The density near ECL2 resolves as two water molecules in our 2.89 Å cryo-EM structure.

Appendix Table S1| EC_{50} values for AKG, ITA, SUC, MA activating to wild-type OXGR1 (related to Fig. 1).

Calcium assay									
**OXGR1**	**AKG**		**ITA**		**SUC**		**MA**		**Surface Expression (WT%)**
	EC_{50} (μM)	E_{max} (WT%)	EC_{50} (μM)	E_{max} (WT%)	EC_{50} (μM)	E_{max} (WT%)	EC_{50} (μM)	E_{max} (WT%)	
**WT**	36±7	100±11	83±8	117±4	480±40	154±4	1200±110	150±3	100±8

All responses were normalized to the maximal response elicited by AKG (100%). Data are mean ± S.E.M. from three independent experiments ($n = 3$), with three technical replicates performed for each experiment. Three independent measurements refer to three different transfections.

Appendix Table S2| Cryo-EM data collection, refinement, and validation statistics. Statistics are provided for the OXGR1-AKG-G_q and OXGR1-ITA-G_q complexes, as well as the corresponding receptor-focused refinements (related to **Figure 2 and Appendix Figure S2**)

**Data Collection**		
**Protein**	**AKG-OXGR1- G_q (OXGR1 focused refined)**	**ITA-OXGR1- G_q (OXGR1 focused refined)**
**Voltage (kV)**	300	300
**Detector**	K3	K3
**Pixel size (Å)**	0.82	0.82
**Defocus range (μm)**	1.0-2.0	1.0-2.0
**Electron dose (e^-/Å^2)**	50	50
**3D Reconstruction**		
**Final Particle number**	405,687	405,209
**Symmetry**	C1	C1
**Overall resolution (Å)**	2.66 (2.89)	2.65 (2.90)
**Model refinement**		
**Model composition**		
**Chains**	7 (3)	7 (3)
**Water**	(2)	(2)
**Ligands**	1	1
**Non-hydrogen atoms**	9,870 (2,309)	9,840 (2,309)
**Protein residues**	1,264 (286)	1,264 (286)
**Bonds (RMSD)**		
**Length (Å)**	0.003	0.003
**Angles (°)**	0.547 (0.561)	0.596 (0.616)
**Ramachandran plot [illegible]**		
**Outliers**	0.00	0.00
**Allowed**	1.53 (1.76)	1.77 (2.11)
**Favored**	98.47 (98.24)	98.23 (97.89)
**Rotamer outlier (%)**	0.37 (0.38)	0.19 (0.38)
**MolProbity score**	1.18 (1.40)	1.31 (1.22)
**Clash score**	3.93 (7.25)	5.64 (4.06)

Note: Data in parentheses represent the results of local and focused refinements performed on the OXGR1 receptor region of each complex. As detailed in the Methods and Results, this focused approach significantly improved the visualization of ligand densities, side-chain conformations, and coordinated water molecules within the orthosteric pocket.

Appendix Table S3| Cryo-EM data collection, refinement, and validation statistics. Statistics are provided for the OXGR1-MA-G_q and OXGR1-SUC-G_q complexes, as well as the corresponding receptor-focused refinements (related to **Figure 2** and **Appendix Figure S3**)

**Data Collection**		
**Protein**	**MA-OXGR1-G_q (OXGR1 focused refined)**	**SUC-OXGR1-G_q (OXGR1 focused refined)**
**Voltage (kV)**	300	300
**Detector**	K3	Falcon 4
**Pixel size (Å)**	0.82	0.73
**Defocus range (µm)**	1.0-2.0	1.0-2.0
**Electron dose ($e^-/Å^2$)**	50	50
**3D Reconstruction**		
**Final Particle number**	436,647	360,330
**Symmetry**	C1	C1
**Overall resolution (Å)**	2.73 (2.97)	2.70 (2.64)
**Model refinement**		
**Model composition**		
**Chains**	7 (3)	7 (3)
**Water**	(4)	(3)
**Ligands**	1	1
**Non-hydrogen atoms**	9,915 (2,356)	9,826 (2,306)
**Protein residues**	1,270 (292)	1,264 (286)
**Bonds (RMSD)**		
**Length (Å)**	0.005 (0.003)	0.004 (0.005)
**Angles (°)**	0.964 (0.589)	0.955 (1.024)
**Ramachandran plot (%)**		
**Outliers**	0.00	0.00
**Allowed**	2.24 (2.76)	1.45 (1.06)
**Favored**	97.76 (97.24)	98.55 (98.94)
**Rotamer outlier (%)**	0.18 (0.37)	0.47 (0.76)
**MolProbity score**	1.31 (1.44)	1.17 (1.23)
**Clash score**	4.88 (5.45)	3.85 (4.49)

Note: Data in parentheses represent the results of local and focused refinements performed on the OXGR1 receptor region of each complex. As detailed in the Methods and Results, this focused approach significantly improved the visualization of ligand densities, side-chain conformations, and coordinated water molecules within the orthosteric pocket.

Appendix Table S4| EC$_{50}$ values for AKG, ITA, SUC, MA activating to wild-type OXGR1 and its variants (related to Fig. 3 and Appendix Figure S5).

Calcium assay									
**OXGR1**	**AKG**		**ITA**		**SUC**		**MA**		**Surface Expression (WT%)**
	EC$_{50}$ (μM)	E$_{max}$ (WT%)	EC$_{50}$ (μM)	E$_{max}$ (WT%)	EC$_{50}$ (μM)	E$_{max}$ (WT%)	EC$_{50}$ (μM)	E$_{max}$ (WT%)	
**WT**	49±20	100±2	43±8	100±10	800±110	100±4	2000±400	100±4	100±8
**N28$^{N\text{-}term}$A**	39±6	92±2	120±30	100±8	300±50	94±0.8	580±30	120±2	90±0.9
**L36$^{1.35X35}$A**	NA[a]	-6.7±1	NA	6.5±8=	NA	6.1±0.2	NA	10±3	21±3
**Y40$^{1.39X39}$A**	NA	3.0±6	NA	6.9±9	NA	-1.5±2	NA	-1.9±6	72±7
**Y82$^{2.53X53}$A**	>1000	31±5	>1000	56±7	NT[b]	NT	NT	NT	83±6
**L89$^{2.60X60}$A**	NA	-6.3±3	NA	-4.5±2.0	NA	2.8±0.9	NA	6.9±1	94±7
**Y93$^{2.64X64}$A**	NA	-4.6±2	NA	3.3±2	NA	5.5±3	NA	17±20	67±5
**E97^{ECL1}A**	>1000	47±0.3	>1000	47±3	1580±260	74±6	6100±1000	79±7	89±5
**R110$^{3.29X29}$A**	NA	-0.74±1	NA	6.5±2	NA	7.0±1	NA	-4.4±2	110±4
**R110$^{3.29X29}$E**	NA	-3.6±0.5	NA	-2.0±3	NA	8.9±5	>10000	36±30	120±4
**R110$^{3.29X29}$Q**	NA	12±6	NA	-2.8±0.7	NA	1.7±2	NA	14±5	120±4
**F113$^{3.32X32}$A**	NA	1.4±2	>1000	26±3	NA	-0.39±2	NA	5.1±10	92±5
**F113$^{3.32X32}$L**	NA	-4.7±0.4	NA	3.7±5	NA	11±1	NA	7.5±3	91±3
**F113$^{3.32X32}$W**	8.3±0.7	85±8	21.3±2	86±2	760±210	82±5	1000±370	99±14	96±6
**H114$^{3.33X33}$A**	NA	-0.29±4	NA	-4.6±2	3600±600	84±4	4200±300	79±4	90±3
**H114$^{3.33X33}$L**	NA	-6.0±2	NA	-5.6±5	NA	1.9±3	NA	-4.4±2	100±6
**L117$^{3.36X36}$A**	630±40	57±0.9	500±20	63±4	NT	NT	NT	NT	85±7
**I121$^{3.40X40}$A**	NA	23±4	NA	15±5	NT	NT	NT	NT	91±5
**L124$^{3.43X43}$A**	75±10	75±7	>1000	36±3	NT	NT	NT	NT	79±7
**F130$^{3.49X49}$E**	NA	-3.4±2	NA	-5.3±3	NT	NT	NT	NT	120±4
**F130$^{3.49X49}$D**	NA	-6.3±1	NA	-5.6±4	NT	NT	NT	NT	120±4
**M168$^{4.60X61}$I**	45±20	85±5	510±60	100±8	1100±60	88±2	3200±300	71±0.9	110±4
**I172$^{4.64X65}$A**	NA	-7.4±2	NA	-0.93±2	NA	0.013±2	NA	11±2	150±3
**R177^{ECL2}A**	180±9	62±8	320±90	110±3	370±70	89±6	1500±300	130±8	92±2
**D185$^{45.54X52}$A**	NA	-2.0±3	NA	4.9±2	NA	-4.4±9	NA	11±3	140±3
**L186^{ECL2}A**	NA	3.2±4	NA	-4.0±2	NA	-0.43±1	NA	14±2	120±3
**L186^{ECL2}F**	74±10	77±5	180±50	80±9	770±60	75±5	>10000	58±4	110±2
**F250$^{6.44X44}$A**	NA	5.8±4	NA	2.2±3	NT	NT	NT	NT	110±10
**F254$^{6.48X48}$A**	NA	-8.4±2	NA	0.87±3	NT	NT	NT	NT	160±10
**F257$^{6.51X51}$A**	NA	10±3	52±10	54±2	>10000	29±1	NA	6.6±7	61±6
**F257$^{6.51X51}$Y**	110±10	82±4	130±40	120±10	81±4	76±2	1900±300	93±2	92±3
**H258$^{6.52X52}$A**	NA	-2.4±5	NA	3.0±6	NT	NT	NT	NT	110±10
**R261$^{6.55X55}$A**	NA	-1.8±2	NA	6.7±2	NT	NT	NT	NT	77±7
**R264$^{6.58X58}$A**	>1000	34±5	>1000	24±3	>10000	57±5	>10000	54±5	84±2
**R268^{ECL3}A**	NA	-11±4	NA	-4.3±4	NA	6.3±4	NA	22±4	74±1

**H281$^{7.32X31}$A**	350±40	84±7	670±60	74±3	4500±1300	87±10	6100±600	67±4	84.3±2
**Y284$^{7.35X34}$A**	NA	10±3	NA	-9±2	NA	4.1±2	NA	4.8±4	98±6
**I285$^{7.36X35}$A**	>1000	22±1	>1000	34±5	NT	NT	NT	NT	63±5
**R288$^{7.39X38}$A**	NA	12±1	NA	11±7	NA	-9.3±4	NA	-2.1±4	86±6
**R288$^{7.39X38}$E**	NA	10±3	NA	24±4	NA	-16±0.6	NA	17±6	160±9
**R288$^{7.39X38}$Q**	>1000	59±5	NA	14±1	NA	11±0.4	NA	16±2	130±6
**L299$^{7.50X50}$P**	NA	-1.5±1	NA	-4.0±9	NA	10.5±3	NA	7.8±3	68±2

[a]NA means not detected because that the activation level is too low to determine EC_{50} values.
[b]NT, Not tested.

All responses of each ligand were normalized to the maximal response elicited by WT (100%). Data are mean ± S.E.M. from three independent experiments ($n = 3$), with three technical replicates performed for each experiment. Three independent measurements refer to three different transfections.

Appendix Table S5| EC_{50} values for AKG, ITA, SUC, MA activating to wild-type SUCR1 and its variants (related to Fig. 5 and Appendix Figure S8).

SUCR1	Calcium assay								Surfac Express (WT%
	SUC		AKG		ITA		MA		
	EC_{50} (μM)	E_{max}[a] (%SUC)	EC_{50} (μM)	E_{max} (%SUC)	EC_{50} (μM)	E_{max} (%SUC)	EC_{50} (μM)	E_{max} (%SUC)	
**WT**	45±10	100±0.5	NA[b]	3.5±3	>1000	26±1	210±70	89±1	100±5
**F72$^{2.53X53}$Y**	1800±300	100±10	NA	4.1±2	NA	3.0±3	NT[c]	NT	120±6
**L102$^{3.32X32}$F**	230±10	100±6	NA	-0.11±2	41±10	74±6	NT	NT	100±3
**I157$^{4.60X61}$M**	480±200	100±7	NA	0.43±0.8	NA	-0.44±1	NT	NT	93±4
**F175^{ECL2}L**	18±3	100±6	NA	3.5±1	340±300	49±2	NT	NT	82±3
**Y248$^{6.51X51}$F**	810±90	100±8	NA	4.7±0.4	NA	15±2	NT	NT	79±6
**F285$^{7.43X42}$A**	3600±800	100±6	NA	1.7±3	NA	6.3±3	NT	NT	71±3
**F285$^{7.43X42}$A-L102$^{3.32X32}$F**	460±80	100±6	NA	5.5±1	33±2	59±1	NT	NT	75±3
**F175^{ECL2}L-I157$^{4.60X61}$M**	150±9	100±1	NA	-6.6±3	NA	-3.1±4	NT	NT	97±5
**2 $^{2.53X53}$Y-L102$^{3.32X32}$F-F285$^{7.43X42}$A**	3900±1300	100±6	NA	5.0±1	>1000	43±5.0	NT	NT	90±3
**02$^{3.32X32}$F-F285$^{7.43X42}$A-Y248$^{6.51X51}$F**	2200±220	100±5	NA	5.7±0.6	>1000	44±0.6	NT	NT	82±3
**2 $^{2.53X53}$Y- F285$^{7.43X42}$A-Y248$^{6.51X51}$F**	2700±780	100±15	NA	19±2	NA	2.2±2	NT	NT	95±8
**L102$^{3.32X32}$F- F175^{ECL2}L**	240±40	100±7	NA	12±3	31±2	72±4	NT	NT	95±3

[a]E_{max} relative to the SUC.

[a]NA means not detected because that the activation level is too low to determine EC_{50} values.

[b]NT, Not tested.

Data are mean ± S.E.M. from three independent experiments ($n = 3$), with three technical replicates performed for each experiment. Three independent measurements refer to three different transfections.

Appendix Table S6| System setup for MD simulations.

Setup item	OXGR-ITA	OXGR-ITA-F130D	OXGR-AKG	OXGR-SUC	OXGR-MA
**Simulation box dimensions**	71.6×72.0×96.1 $Å^3$	72.8×73.4×94.2 $Å^3$	72.0×71.8×95.9 $Å^3$	75.04×74.84×93.4 $Å^3$	74.6×73.6×95.5 $Å^3$
**Total atom number**	40,637	42,256	41,359	44,529	43,087
**Total water number**	5,696	5,968	5,977	6,301	6,142
**Lipid (POPC) number**	98	102	95	109	103
