# Supplementary figures and images for: Molecular architecture of OXGR1 reveals an evolutionary conserved mechanisms for metabolite surveillance
Source: EMBO J. 2026 Jun 3;45(14):4931–55. doi: 10.1038/s44318-026-00823-y (PMC13372822; doi:10.1038/s44318-026-00823-y)

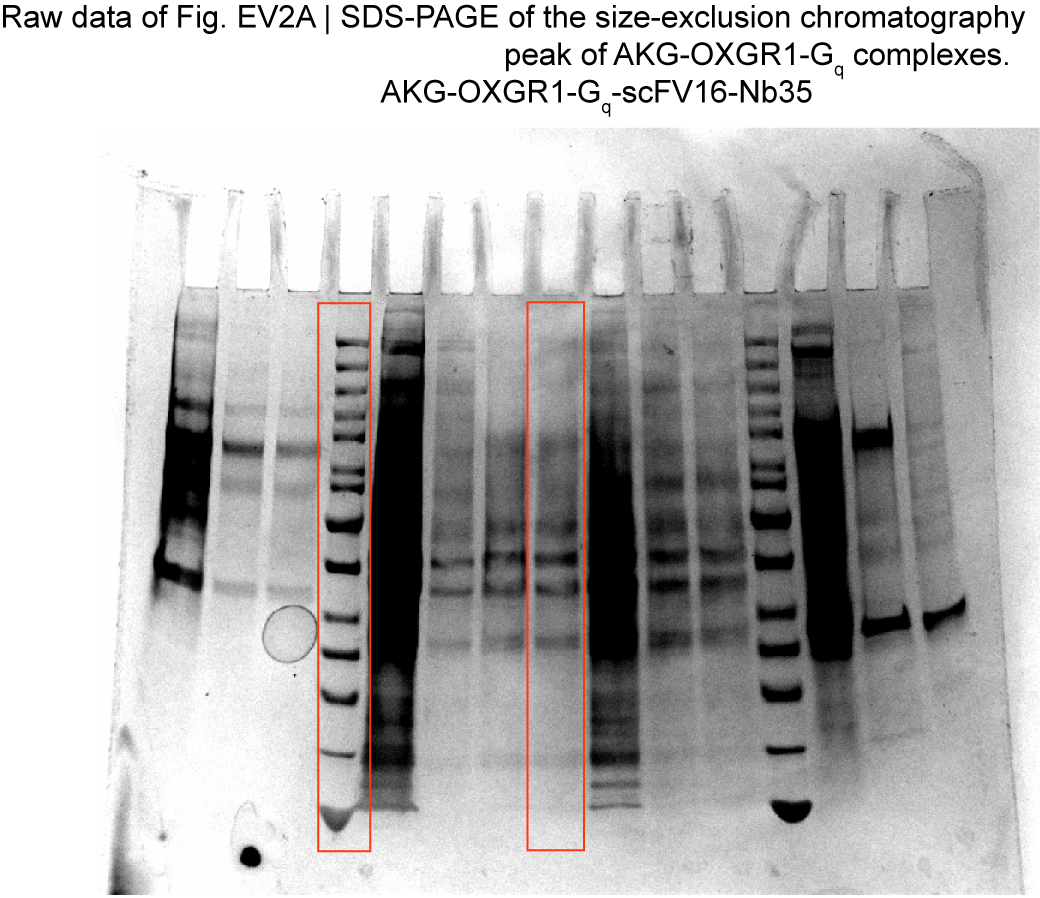

Supplement: Supplementary file 9 — Appendix Figure Source Data [file 44318_2026_823_MOESM9_ESM.zip › Appendix Figure Source Data/Appendix Figure S2/S2A/S2A-uncropped SDS-PAGE AKG-OXGR1-Gq.tif]

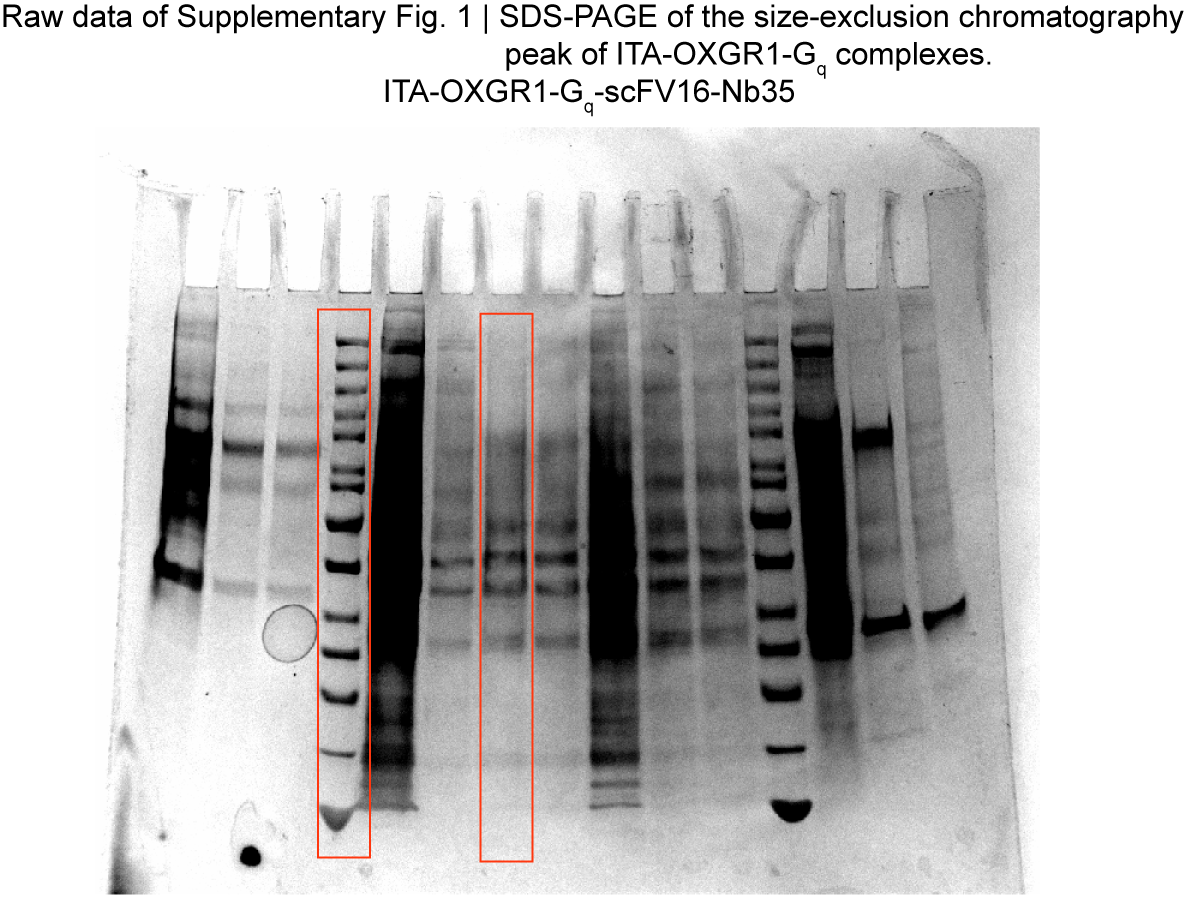

Supplement: Supplementary file 9 — Appendix Figure Source Data [file 44318_2026_823_MOESM9_ESM.zip › Appendix Figure Source Data/Appendix Figure S2/S2H/S2H-uncropped SDS-PAGE ITA-OXGR1-Gq.tif]

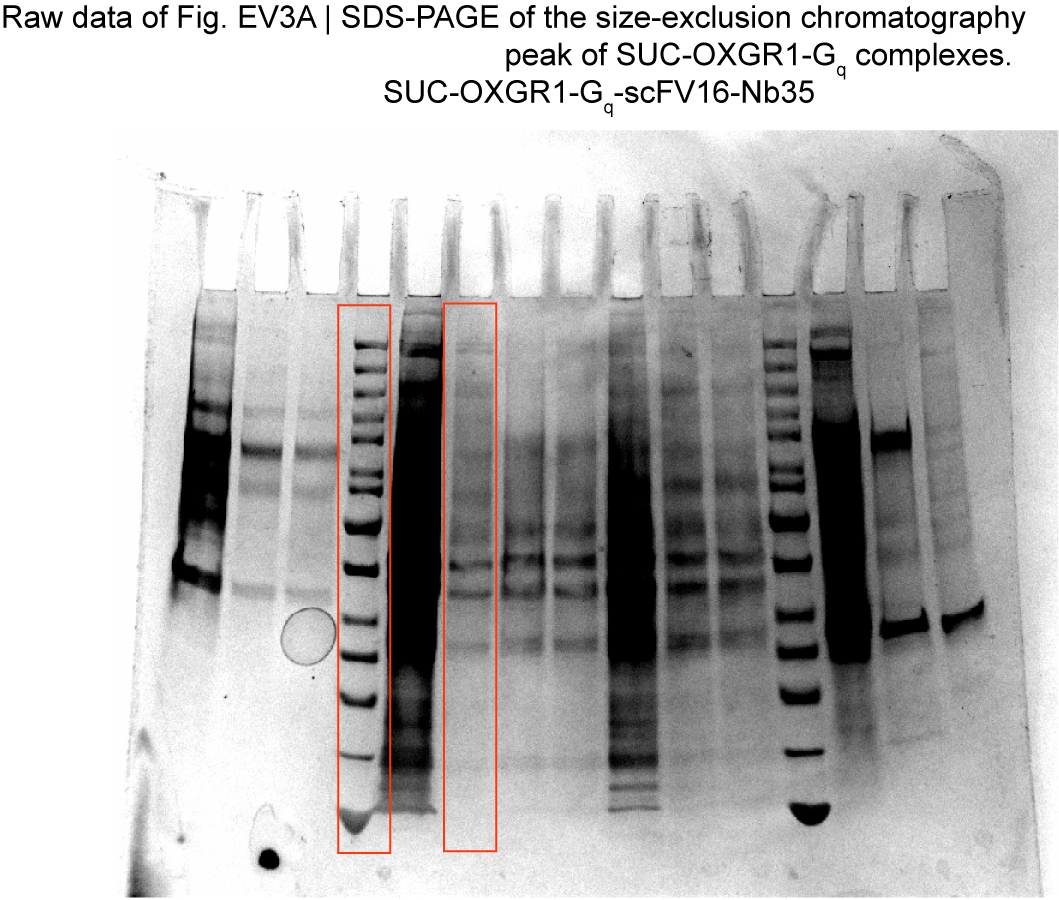

Supplement: Supplementary file 9 — Appendix Figure Source Data [file 44318_2026_823_MOESM9_ESM.zip › Appendix Figure Source Data/Appendix Figure S3/S3A/S3A-uncropped SDS-PAGE SUC-OXGR1-Gq.tif]

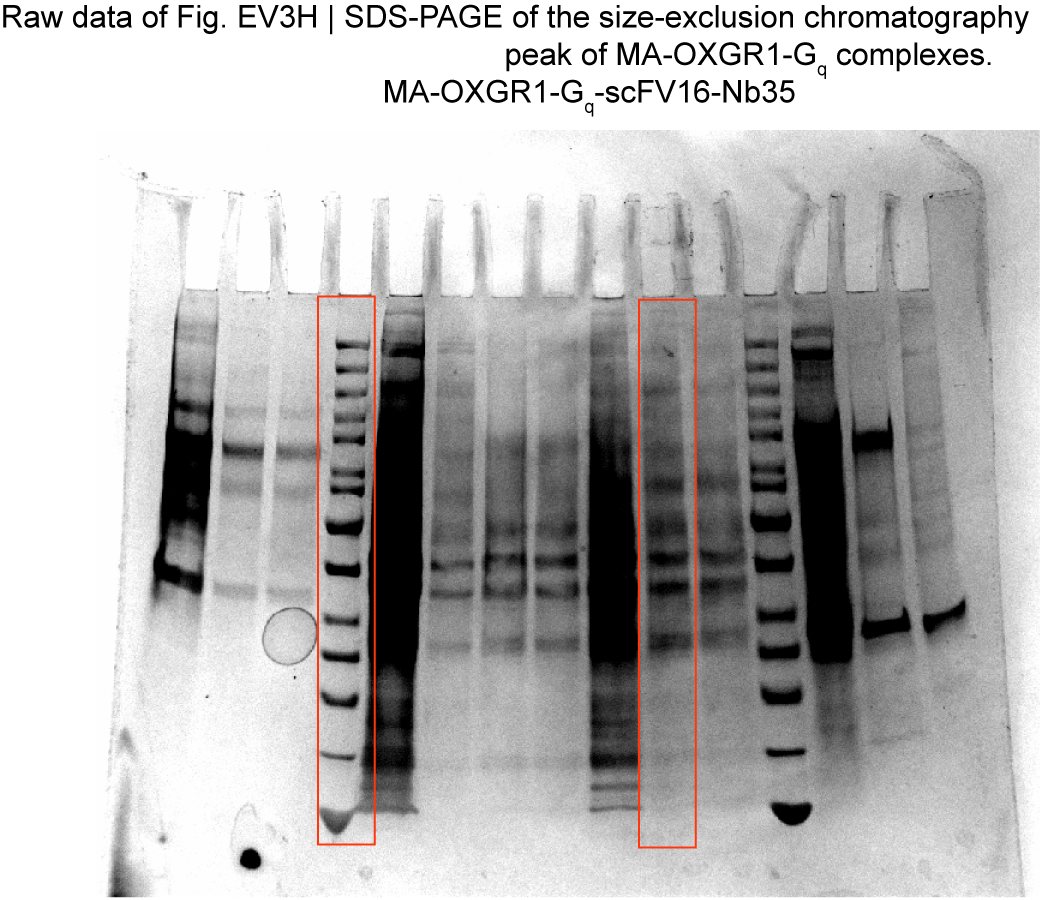

Supplement: Supplementary file 9 — Appendix Figure Source Data [file 44318_2026_823_MOESM9_ESM.zip › Appendix Figure Source Data/Appendix Figure S3/S3H/S3H-uncropped SDS-PAGE MA-OXGR1-Gq.tif]
