# Supplementary material for: Molecular architecture of OXGR1 reveals an evolutionary conserved mechanisms for metabolite surveillance
Source: EMBO J. 2026 Jun 3;45(14):4931–55. doi: 10.1038/s44318-026-00823-y (PMC13372822; doi:10.1038/s44318-026-00823-y)
Supplement: Supplementary file 10 — Expanded View Figures [file 44318_2026_823_MOESM10_ESM.pdf]

Expanded View Figures

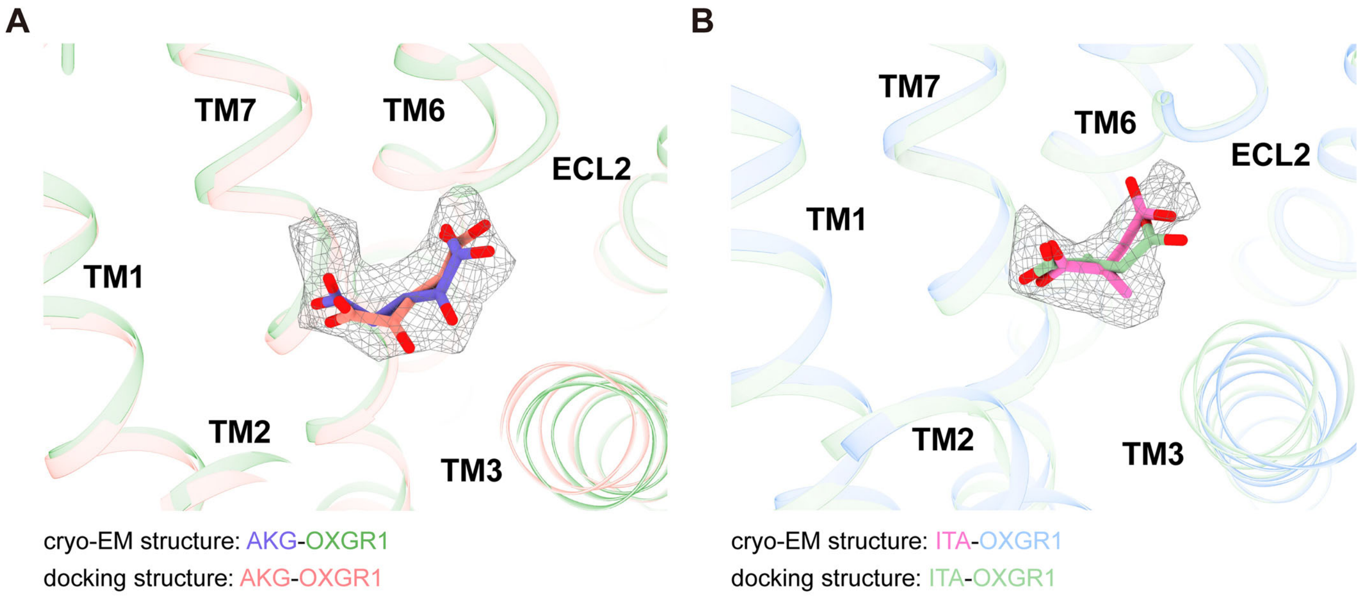

Figure EV1. Structural validation of four ligand-binding modes in OXGR1 by molecular docking and cryo-EM density maps.

Comparison of AKG (**A**), ITA (**B**), SUC (**C**), and MA (**D**) binding postures in molecular docking and structural modeling of OXGR1. Each ligand is aligned with the corresponding cryo-EM density represented in mesh, validating the proposed binding conformations. The densities of respective ligands have been extracted from their local structures of receptors and shown in surface presentation. All ligand density maps are shown at a contour level of 0.3, and the local resolutions of AKG/ITA/SUC/MA-bound OXGR1 are 2.89 Å, 2.90 Å, 2.70 Å, 2.97 Å, respectively.

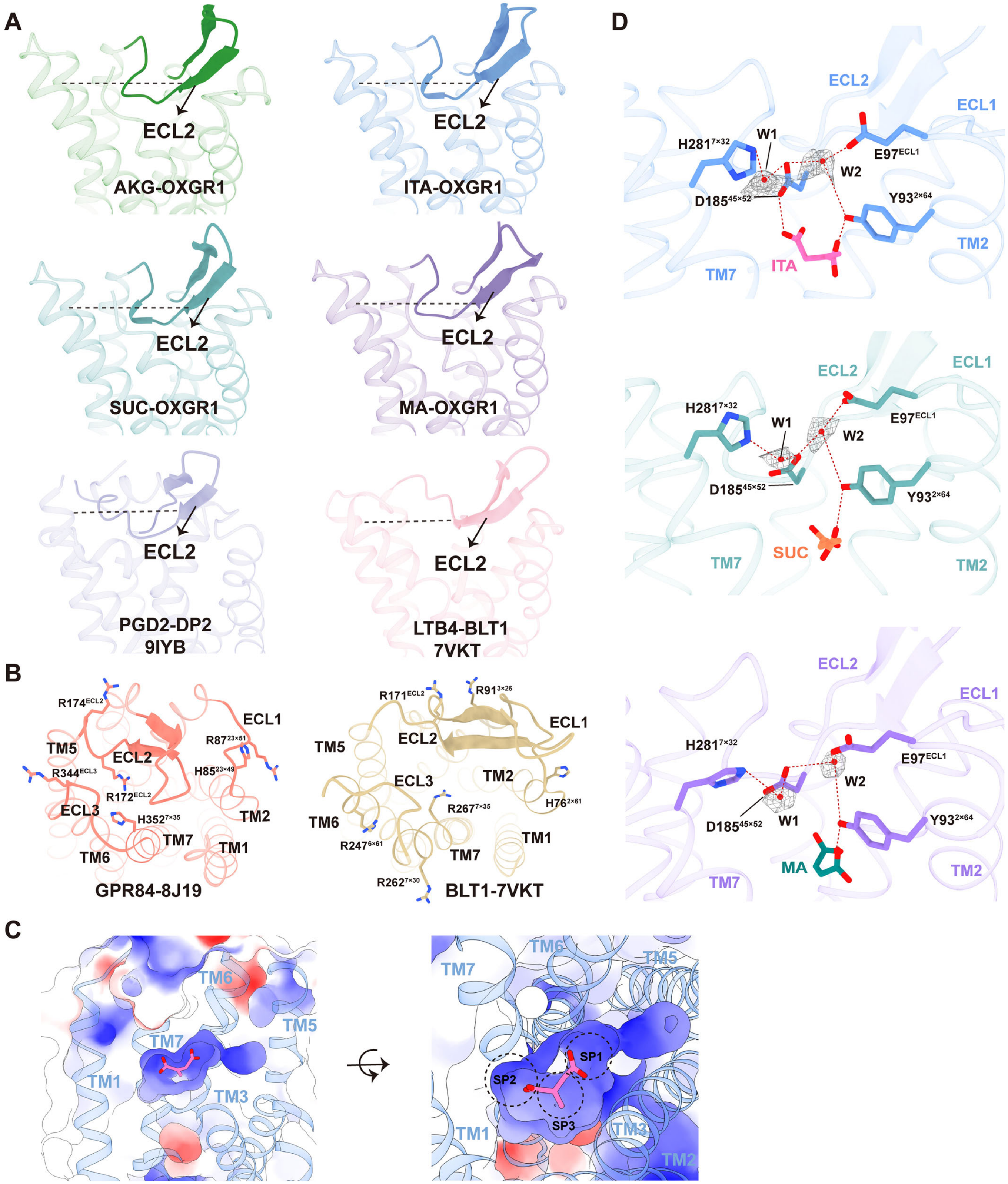
A
ECL2
AKG-OXGR1
ECL2
ITA-OXGR1
ECL2
SUC-OXGR1
ECL2
MA-OXGR1
ECL2
PGD2-DP2
9IYB
ECL2
LTB4-BLT1
7VKT
B
R174 ECL2
ECL1
TM5
ECL2
R87 23×51
R344 ECL3
H85 23×49
R172 ECL2
ECL3
H352 7×35
TM2
TM7
TM1
TM6
GPR84-8J19
R171 ECL2
R91 3×26
ECL1
TM5
ECL2
ECL3
TM2
H76 2×61
R267 7×35
TM6
R247 6×61
TM1
TM7
R262 7×30
BLT1-7VKT
C
TM6
TM5
TM7
TM1
TM3
TM6
TM7
TM5
SP1
SP2
SP3
TM1
TM3
TM2
D
ECL2
ECL1
H281 7×32
W1
E97 ECL1
W2
D185 45×52
Y93 2×64
TM2
ITA
TM7
ECL2
ECL1
H281 7×32
W1
E97 ECL1
W2
D185 45×52
Y93 2×64
SUC
TM7
TM2
ECL2
ECL1
H281 7×32
E97 ECL1
W2
D185 45×52
W1
Y93 2×64
MA
TM7
TM2

Figure EV2. Structural features of OXGR1-G_q complex.

(**A**) A similar structure feature of ECL2-β-hairpin partially buried within the orthosteric pocket. (**B**) The structures of BLT1 and GPR84 reveal a convergent "cationic lure" ligand entry mechanism featuring, as observed in the case of OXGR1 in Fig. 2E,F. (**C**) The ligand-binding pocket, with ITA-OXGR1 as an additional description, is characterized by a polar and positively charged nature. (**D**) An additional polar hydrogen-bond network in ITA/SUC/MA-OXGR1 structures mediated by two water molecules, W1 and W2, is surrounded by key residues shown in sticks. The ordered water molecules (W1/W2) are overlaid with their corresponding cryo-EM density represented in mesh, and their density maps are shown at a contour level of 0.35. Key polar interactions are shown with red dashed lines.

© The Author(s)

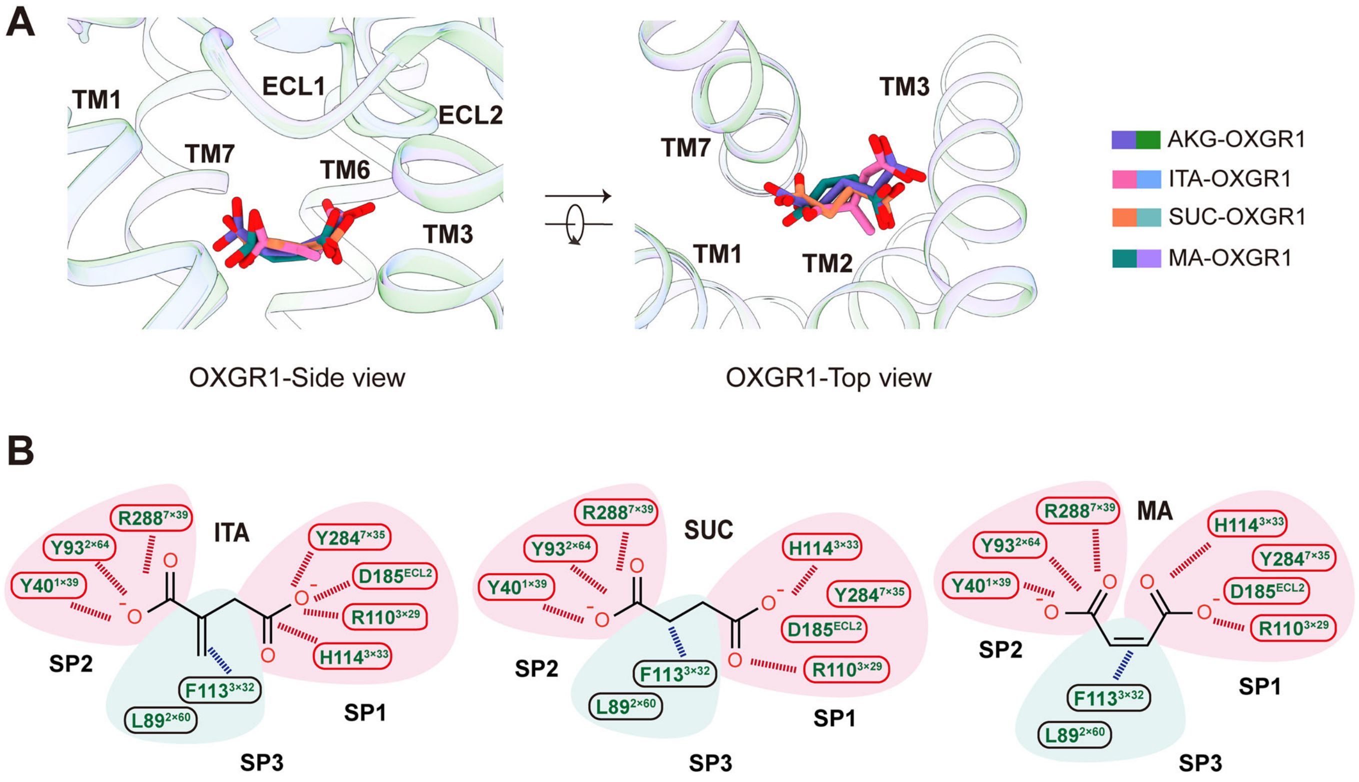

Figure EV3. Structural comparison of ligand-binding modes in OXGR1.

(**A**) Superimposed views of the four ligand-bound OXGR1 structures. These top and side views demonstrate that all four dicarboxylate ligands AKG, ITA, SUC, and MA occupy a similar location within the orthosteric pocket and adopt a consistently horizontal orientation. (**B**) The interaction ligplot style diagrams for the other three ligands (ITA, SUC, MA). Key polar interactions in the pink region are shown with red dashed lines and red circles, and hydrophobic interactions in the cyan region are shown with blue dashed lines and black circles. AKG/ITA engage in direct polar contacts with D18545$^{\times 52}$ and Y284$^{7\times 3}$, whereas these contacts are absent in SUC/MA.

© The Author(s)

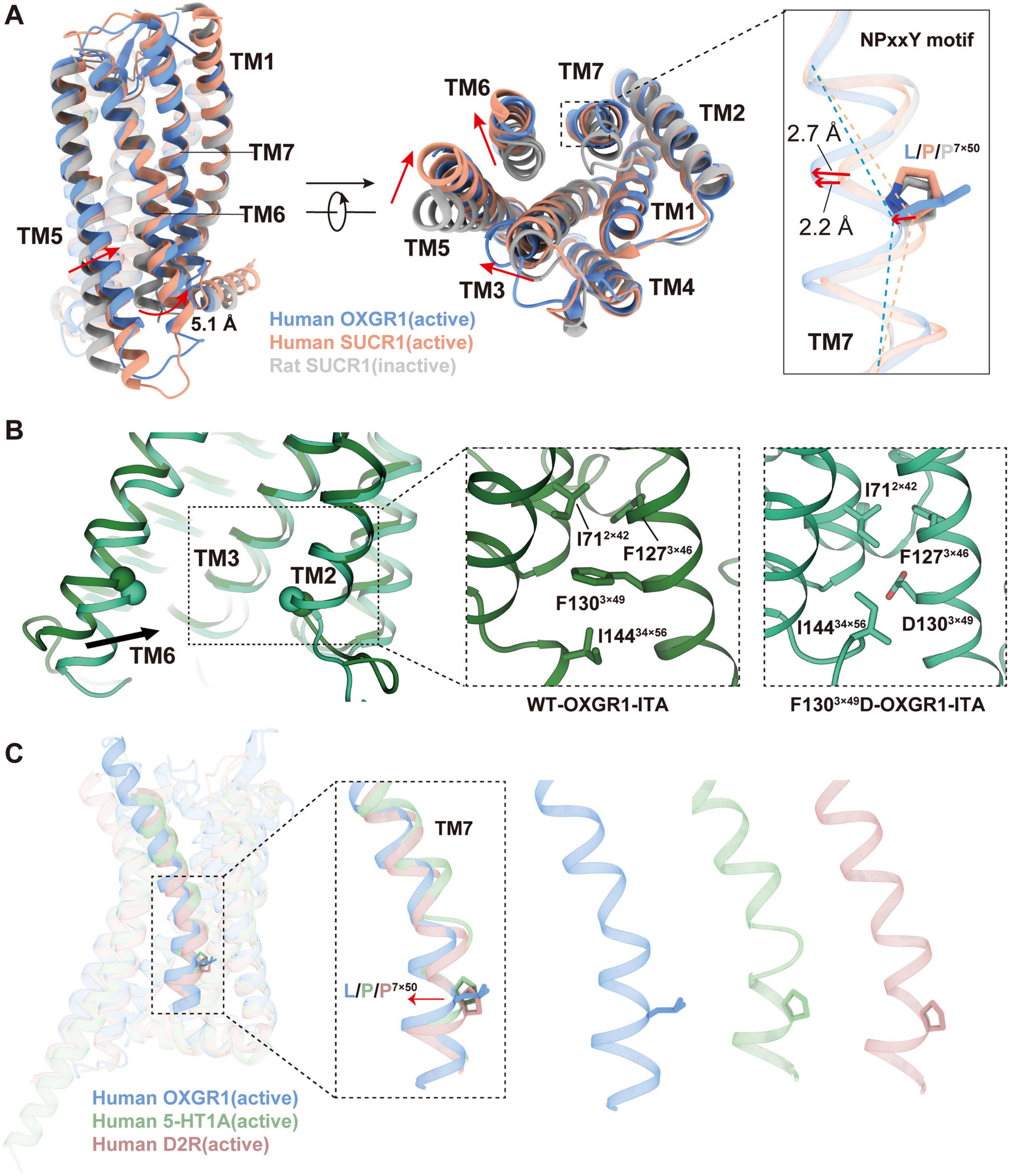
A
TM1
TM7
TM6
TM5
5.1 Å
TM7
TM6
TM2
TM1
TM5
TM3
TM4
Human OXGR1(active)
Human SUCR1(active)
Rat SUCR1(inactive)
NPxxY motif
2.7 Å
2.2 Å
L/P/P7×50
TM7
B
TM3
TM2
TM6
I71 2×42
F127 3×46
F130 3×49
I144 34×56
WT-OXGR1-ITA
I71 2×42
F127 3×46
I144 34×56
D130 3×49
F130 3×49 D-OXGR1-ITA
C
TM7
L/P/P7×50
Human OXGR1(active)
Human 5-HT1A(active)
Human D2R(active)

Figure EV4. Unique activation motifs contribute to OXGR1 signaling.

(**A**) Distinct conformational changes in OXGR1 activation. Structural comparison of the ITA-bound OXGR1 with the inactive and active SUCR1 revealed a pronounced outward displacement of the cytoplasmic end of TM6, a rotation of TM5, as well as the inward movement of TM7, all hallmark features of receptor activation. (**B**) FRY motif drives hydrophobic pocket remodeling and outward movement of TM6. Inward movement of TM6 in F130$^{3\times49}$D system is observed in comparison with WT system, and the zoom-in view of the hydrophobic pocket around F/D$^{3\times49}$ reveals that OXGR1's FRY motif stabilizes the active state through enhanced hydrophobic packing. The present findings indicate that the FRY motif of OXGR1 facilitates its activation. (**C**) Structural comparison of OXGR1 with 5-HT$_{1A}$ and D2R highlights OXGR1's divergent rearrangement in the NPxxY motif, which differs from the canonical Class A GPCR mechanisms.

© The Author(s)

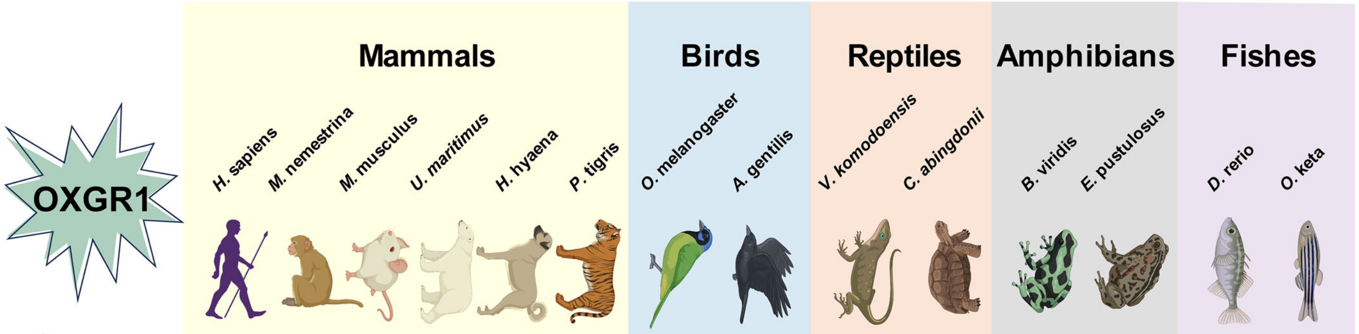

		H. sapiens	M. nemestrina	M. musculus	U. maritimus	H. hyaena	P. tigris	O. melanogaster	A. gentilis	V. komodoensis	C. abingdonii	B. viridis	E. pustulosus	D. rerio	O. keta
Ligand Recognition	$Y40^{1\times39}$	Y	Y	Y	Y	Y	Y	Y	Y	Y	Y	Y	Y	Y	Y
	$Y93^{2\times64}$	Y	Y	Y	Y	Y	Y	Y	S	Y	Y	Y	Y	Y	Y
	$R110^{3\times29}$	R	R	R	R	R	R	R	R	R	R	R	R	R	R
	$F113^{3\times32}$	F	F	F	F	F	F	F	F	F	F	F	F	F	F
	$H114^{3\times33}$	H	H	H	H	H	H	Y	Y	H	H	H	H	Y	H
	$D185^{45\times52}$	D	D	D	D	D	D	D	D	D	D	D	D	D	D
	$R288^{7\times39}$	R	R	R	R	R	R	K	K	R	R	R	R	R	R
Activation	$Y82^{2\times53}$	Y	Y	Y	Y	Y	Y	Y	Y	Y	Y	H	H	Y	Y
	$F113^{3\times32}$	F	F	F	F	F	F	F	F	F	F	F	F	F	F
	$L117^{3\times36}$	L	L	L	L	L	L	L	M	L	L	L	L	L	L
	$I121^{3\times40}$	I	I	I	I	I	I	I	I	I	I	I	I	I	I
	$F254^{6\times48}$	F	F	F	F	F	F	F	F	F	F	F	F	F	F
	$F257^{6\times51}$	F	F	F	F	F	F	F	F	F	F	L	L	Y	Y
NLxxY motif	$N298^{7\times49}$	N	N	N	N	N	N	N	N	N	N	N	N	N	N
	$L299^{7\times50}$	L	L	L	L	L	L	L	L	L	L	L	L	L	L
	$Y302^{7\times53}$	Y	Y	Y	Y	Y	Y	Y	Y	Y	Y	Y	Y	Y	Y
FRY motif	$F130^{3\times49}$	F	F	F	F	F	F	F	F	F	F	F	F	F	F
	$R131^{3\times50}$	R	R	R	R	R	R	R	R	R	R	R	R	R	R
	$Y132^{3\times51}$	Y	Y	Y	Y	Y	Y	Y	Y	Y	Y	Y	Y	F	F

Figure EV5. Evolutionary conservation of OXGR1 function in vertebrates.

Sequence alignment of key residues involved in the ligand recognition, activation mechanism, $F^{3\times49}$-$R^{3\times50}$-$Y^{3\times51}$ motif (D/ERY motif in common GPCRs), and N-$L^{7.50}$-xx-$Y^{7.53}$ motifs (NPxxY motif in common GPCRs) of OXGR1 among different species in vertebrates. Highlight basic amino acids in blue, acidic amino acid in red, and other residues in black. The animal images were generated using BioRender (https://biorender.com).

© The Author(s)
