# Supplementary material for: Molecular architecture of OXGR1 reveals an evolutionary conserved mechanisms for metabolite surveillance
Source: EMBO J. 2026 Jun 3;45(14):4931–55. doi: 10.1038/s44318-026-00823-y (PMC13372822; doi:10.1038/s44318-026-00823-y)
Supplement: Supplementary file 2 — Peer Review File [file 44318_2026_823_MOESM2_ESM.pdf]

THE EMBO JOURNAL

Molecular architecture of OXGR1 reveals an evolutionary conserved mechanism for metabolite surveillance

Xinyue zhang, Yujie lu, Xinheng He, Shimeng guo, Changyao li, Yu wang, Yuan Gao, Juxia yao, Qingning yuan, Yinshan tang, Jing hu, Wen Hu, Zijuan Luo, Kai Wu, Yue wang, Wanchao yin, Xin xie, Huaqiang Xu, and Heng Liu

Corresponding author(s): Heng Liu (liuheng@simm.ac.cn) , Huaqiang Xu (eric.xu@simm.ac.cn), Xin xie (xxie@simm.ac.cn)

Review Timeline:

Submission Date:	28th Sep 25
Editorial Decision:	17th Dec 25
Revision Received:	26th Jan 26
Editorial Decision:	11th Mar 26
Revision Received:	19th Mar 26
Accepted:	5th May 26

Editor: Cornelius Schneider

Transaction Report:

(Note: With the exception of the correction of typographical or spelling errors that could be a source of ambiguity, letters and reports are not edited. Depending on transfer agreements, referee reports obtained elsewhere may or may not be included in this compilation. Referee reports are anonymous unless the Referee chooses to sign their reports.)

1st Editorial Decision **17th Dec 2025**

Dear Dr. Liu,

Thank you for submitting your manuscript for consideration by the EMBO Journal. It has now been seen by three referees whose comments are shown below.

Given the referees' positive recommendations, I would like to invite you to submit a revised version of the manuscript, addressing the comments of all three reviewers. I should add that it is EMBO Journal policy to allow only a single round of revision, and acceptance of your manuscript will therefore depend on the completeness of your responses in this revised version.

When preparing your letter of response to the referees' comments, please bear in mind that this will form part of the Review Process File, and will therefore be available online to the community. For more details on our Transparent Editorial Process, please review our Editorial Policies: https://link.springer.com/partners/embo-press/editorial-policies

We generally allow three months as standard revision time. As a matter of policy, competing manuscripts published during this period will not negatively impact on our assessment of the conceptual advance presented by your study. However, we request that you contact the editor as soon as possible upon publication of any related work, to discuss how to proceed. Should you foresee a problem in meeting this three-month deadline, please let us know in advance and we may be able to grant an extension.

Thank you for the opportunity to consider your work for publication. I look forward to your revision.

Yours sincerely,

Cornelius Schneider, PhD
Editor
The EMBO Journal
c.schneider@embojournal.org

Instructions for preparing your revised manuscript:

Read our guidance for manuscript revisions and related editorial policies: https://link.springer.com/journal/44318/submission-guidelines#cms-Revised-submissions

Please make sure you upload a letter of response to the referees' comments together with the revised manuscript.

Please also check that the title and abstract of the manuscript are brief, yet explicit, even to non-specialists.

When assembling figures, please refer to our figure preparation guideline in order to ensure proper formatting and readability in print as well as on screen:
https://media.springernature.com/original/springer-cms/rest/v1/content/27825798/data/v1

At EMBO Press we ask authors to provide source data for the main manuscript figures. You will receive a separate email with instructions for providing source data with your revised manuscript, including how to upload and organize the files.

IMPORTANT: When you send the revision we will require
- a point-by-point response to the referees' comments, with a detailed description of the changes made (as a word file).
- a word file of the manuscript text.
- individual production quality figure files (one file per figure)
- a complete author checklist
- Expanded View files (replacing Supplementary Information)
- a Reagents and Tools Table as part of the Methods section

Please remember: Digital image enhancement is acceptable practice, as long as it accurately represents the original data and conforms to community standards. If a figure has been subjected to significant electronic manipulation, this must be noted in the figure legend or in the 'Materials and Methods' section. The editors reserve the right to request original versions of figures and the original images that were used to assemble the figure.

We realize that it is difficult to revise to a specific deadline. In the interest of protecting the conceptual advance provided by the work, we recommend a revision within 3 months (17th Mar 2026). Please discuss the revision progress ahead of this time with the editor if you require more time to complete the revisions. Use the link below to submit your revision:

https://emboj.msubmit.net/cgi-bin/main.plex

Referee #1:

This is a very staightforward paper describing the sturcture of the itaconate and alphaKG receptor OXGR1. The ligand binding region is uncovered and described in detail. Hyrdophobic residues are identified as determinants of ligand potency and selectivity. Evolutionary conservation is also revealed. Overall I have no issues with this paper and recommend publication.

Referee #2:

This manuscript by Zhang et al., elucidates the cryo-EM structures of OXGR1 in complex with multiple metabolite agonists and G-proteins using state-of-the-art methodologies. The structural snapshots uncover the overall topology of the orthosteric binding pocket with direct implications to understand the molecular determinants of ligand binding and selectivity. They also combine the structural insights with simulation and mutagenesis studies to validate and expand the key findings, especially in terms of distinct conformations and arrangements of the molecular switches in the receptor. Overall, the study is beautifully designed and executed, and presentation is easy to follow with clear and strong message, and I strongly recommend its publication. I have a couple of minor suggestions for the authors to consider during revision.
01. It may be useful for the non-expert readers to show a couple of different views (other than 180{degree sign} as presented in the supplemental figure) of the ligand with the corresponding EM density considering their chemical structures.
02. The MD simulation trajectories should be deposited in the corresponding database as it has become a broadly followed practice in the field.
03. The deviation from DRY and NPxxY motif is very intriguing, and mutagenesis data clearly demonstrate that D/E to F substitution in OXGR1 is functionally critical. The authors may consider testing P to L substitution in the NPxxY motif as well using endocytosis as readout.
04. Overall, the pharmacological assays are done reasonably well but, in some cases, where the response is observed only at the highest concentration e.g. metabolite screening or mutant validation, it could be more convincing to do a control with mock-transfected cells as well.

Arun K. Shukla, PhD

Referee #3:

Zhang et al have determined four cryo-EM structures of G protein-coupled OXGR1 bound to four different dicarboxylic acids SUC, MA, ITA and AKG (succinate, malate, itaconate and alpha-ketoglutarate). These metabolites play an important role in regulation of the metabolic state of cells, so the manuscript is an important step-change in our understanding of how these molecules promote signalling. The four dicarboxylic acids bind in a similar manner in the orthosteric binding pocket with important contributions being made by strong polar interactions with the positively charged residues R110, H114 and R288. A number of potential water molecules were also identified in the density maps. Mutation of these key residues and others involved in binding the ligands prevented or greatly reduced receptor activation. The relative contributions of the residues to binding were explored by molecular dynamics simulations to identify those elements most important for ligand potency. The authors then compare the structures with another GPCR that specifically binds succinate (SUCR1), and in a different manner to OXGR1. The authors elegantly show that they could alter the binding characteristics of SUCR1 through comparison with their OXGR1 structures, so that mutant SUCR1 gained binding of ITA that is normally unable to activate SUCR1. Finally, the authors perform mutagenesis to highlight residues that are involved in receptor activation in a manner analogous to other Class A GPCRs, despite having unique non-canonical motifs such as FRY rather E/DRY. Overall, the manuscript presents a comprehensive structure-function analysis of OXGR1.

The manuscript is very thorough, well-written and amply supported by many clear figures. I have only a few queries and suggestions for clarifications that I have listed in the order they appear in the manuscript.

1. From the introduction I am unclear about where signalling takes place. TCA cycle metabolites are usually thought to be found intracellularly, so does this mean that OXGR1 is on intracellular membranes? Or are the metabolites transported out of the cell, allowing them to signal to cells from the extracellular surface like many other GPCRs? A few sentences addressing this would be very helpful. I appreciate that this becomes obvious when reading the results section and perhaps lines 117-126 would be better

in the introduction
2. Line 137-138. 'Selective coupling to Gq-mediated signalling' implies OXGR1 couples only to Gq, but the authors tested only Gs and Gi and it is conceivable that other untested G proteins could couple. Please re-phrase to avoid introducing this misconception.
3. Please include a figure where the four ligand orientations in the structures are compared after the structures have been aligned. In addition, four ligplot style figures showing the residues involved in binding the respective ligands should be included to improve the clarity of the comparisons made.
4. Lines 209-211: I do not understand this sentence.
5. Lines 244-247: There is 12 kcal/mol difference between the binding free energies for ITA and AKG and yet the EC50s are virtually identical. How can this be? Please explain, especially given that the binding free energies for SUC and MA are virtually identical and so are their EC50s.
6. Line 254: I do not understand what is meant by 'pocket stabilisation'
7. Line 380: delete 'succinate' and 'malate' as the abbreviations have been introduced before
8. Figure 1: In panel B, the structures are given of the protonated forms yet the pKas of the acidic groups are between about 2-5, and therefore would be expected to be unprotonated at pH 7.4 (buffer used for purification). The authors also mention that they anticipate the acids would be unprotonated given the proximity of positively charged residues in the structures. Please could you therefore draw the structures as being unprotonated and show the delocalised electron structures. It would be helpful in the text if the pKas of the acidic groups are mentioned and state they are unprotonated.
9. Figure 1: In panel D it seems strange that all the curves stop at 100% and the curves for SUC and MA have not plateaued. The dicarboxylic acids have high solubility in water so I would have thought it would have been possible to use higher concentrations. In addition, what is the size of the assay window in this experiment? This should be much larger than the window of the recorded response, but if it is limited to 100% then it could explain why all the curves finish at the same level.
10. Figure 1, line 10 and panel C: Please do not use the term NA for 'not active'. In English it is a standard abbreviation for 'not applicable'. I would just delete this and put outlines of boxes for white areas that showed no binding. In addition, just write 'Not tested' in the area where particular combinations were not tried and perhaps colour this solid grey for clarity.
11. In all panels containing plotted data, please state the number of technical replicates per experiment after stating the number of independent experiments e.g. 'three independent experiments were performed in duplicate'. Please also give a link to a table of the numerical data that was plotted, along with EC50s where relevant and their respective errors.
12. Figure 4, panel D: Please define what distance is being measured here e.g. is it between specific atoms (which?) or a centre of mass (COM)?
13. Figure 4, line 78-79. It is difficult to distinguish between the blue and red lines in the figure. Lines 80-82, I cannot see this in the figure.
14. Figures EV2 & EV3: In the local resolution maps (E, L, G, N), please choose appropriate scales for each panel so that everything is not all blue e.g. between 2 Å to 4 Å.
15. Figure EV9. What do the blank circles mean?
16. Tables S1 and S2. Extra columns have been included for the receptor alone after focussed refinement. I am not sure this is necessary and may be confusing. The extra columns imply that they are different structures when in fact the receptor structures are absolutely identical (RMSD 0.000). Please make it clear in the Methods and these tables that the receptor models are the same. Perhaps only have one column and put the important numbers (resolution, number of water molecules) in parentheses for the focussed refined model and give a footnote to the table? In the results section of the main text it would also be worth mentioning the improvement in density that you see on focussed refinement of the receptors (not nearly enough people are doing receptor focussed refinements).

Typos
Line 156: deletion 'portion'
Line 166: insert 'was' after 'arrangement'
Line 195: change to 'the four metabolites AKG, ITA...'
Line 215: do not use possessives (ligands')
Line 238: change to'...different ligand potencies'.

Referee #4:

α-ketoglutarate (AKG) and itaconate (ITA) are important metabolites and OXGR1 plays critical roles in metabolism regulation by sensing AKG and ITA. This manuscript reported the Cryo-EM structures of active state OXGR1 bound with AKG and ITA, as well as the related but weaker binding metabolites succinate (SUC) and maleate (MA) at the 3.0Å higher resolutions. The ligands and the binding environment are clearly identified and are consistent with the results of molecular docking. Systematic mutagenesis and activity assay proved the roles of critical residues in ligand binding and signal transduction. The experimental data are solid and the manuscript is well written. This manuscript provide deep insights into specific ligand recognition and G protein coupling mechanism and may have contribution to future drug design targeted to OXGR1.
Only a concern should be addressed: the density map in Figure 2, Figure EV4 and EV5 look different, especially in figure EV5, although all of the contour levels are 0.3.

Manuscript ID: EMBOJ-2025-122560

Title: Molecular architecture of OXGR1 reveals evolutionary conserved mechanisms for metabolite surveillance

We sincerely thank the editor and all referees for their time, effort, and constructive comments. We are particularly encouraged by the consistent recommendation for publication and the recognition of our work's novelty and completeness. Having addressed all concerns through additional experiments and refined analyses, the manuscript has significantly improved. We are confident that this revised manuscript is now suitable for publication in ***The EMBO Journal***.

In this revision, we have systematically addressed the referees' comments and advice. We expanded the Introduction to clarify how intracellular metabolites are exported via specific transporters to activate plasma-membrane OXGR1. We provided multiple views of each ligand within its EM density to facilitate structural evaluation and standardized this visualization across all relevant Figures. To strengthen our functional conclusions, we performed calcium and immunofluorescence assays for the OXGR1-L299^{7x50}P mutant, demonstrating the critical role of the un-conserved NLxxY motif in OXGR1 activation and endocytosis. These experiments were further supported by mock-transfected negative controls to ensure data rigor. Furthermore, we utilized Epik to confirm the protonation states of all four ligands at pH 7.4, and have deposited all associated molecular dynamics simulation trajectories in public database to support transparency. Finally, we refined the manuscript's text, figure legends, tables, and methodological descriptions to ensure a more cohesive logical flow and overall clarity.

Below, we provide detailed point-by-point responses to each referee. The referees' original comments are shown in **black**, and our responses are in **blue**.

Referee #1:

This is a very straightforward paper describing the structure of the itaconate and alpha-KG receptor OXGR1. The ligand-binding region is uncovered and described in detail. Hydrophobic residues are identified as determinants of ligand potency and selectivity. Evolutionary conservation is also revealed. Overall, I have no issues with this paper and recommend publication.

Response: We are grateful to Referee #1 for the positive evaluation and the strong recommendation for publication.

Referee #2:

This manuscript by Zhang et al., elucidates the cryo-EM structures of OXGR1 in complex with multiple metabolite agonists and G-proteins using state-of-the-art methodologies. The structural snapshots uncover the overall topology of the orthosteric binding pocket with direct implications to understand the molecular determinants of ligand binding and selectivity. They also combine the structural insights with simulation and mutagenesis studies to validate and expand the key findings, especially in terms of distinct conformations and arrangements of the molecular switches in the receptor. Overall, the study is beautifully designed and executed, and the presentation is easy to follow with clear and strong message, and I strongly recommend its publication. I have a couple of minor suggestions for the authors to consider during revision.

Response: We sincerely thank Referee #2 for strongly recommending our manuscript for publication, as well as for highlighting the significance of our work. We have carefully addressed each suggestion to improve the manuscript.

In revision, we have added different views of four ligands with their EM density to facilitate structural evaluation (**Appendix Figures S4B, D, F, H)**. The associated MD simulation trajectories are now publicly available (https://doi.org/10.6084/m9.figshare.28946102.v2). We have also provided calcium assays and immunofluorescence assays for OXGR1-L299^{7x50}P mutant (**Figure. 6B)**, further demonstrating the critical role of the NLxxY motif in OXGR1 activation. Besides, negative control experiments (empty vector) in the functional data have been included to strengthen the rigor of the data.

Below we respond point-by-point.

1. It may be useful for the non-expert readers to show a couple of different views (other than 180 degrees as presented in the supplemental Figure) of the ligand with the corresponding EM density considering their chemical structures.

Response: We thank the referee for this constructive suggestion. In response, we have added supplementary views of the ligand with their corresponding EM density from multiple angles to the revised **Appendix Figures S4B, D, F, H** (also shown in **Figure. R1** below). We believe these views significantly aid in the assessment of the ligand-binding modes.

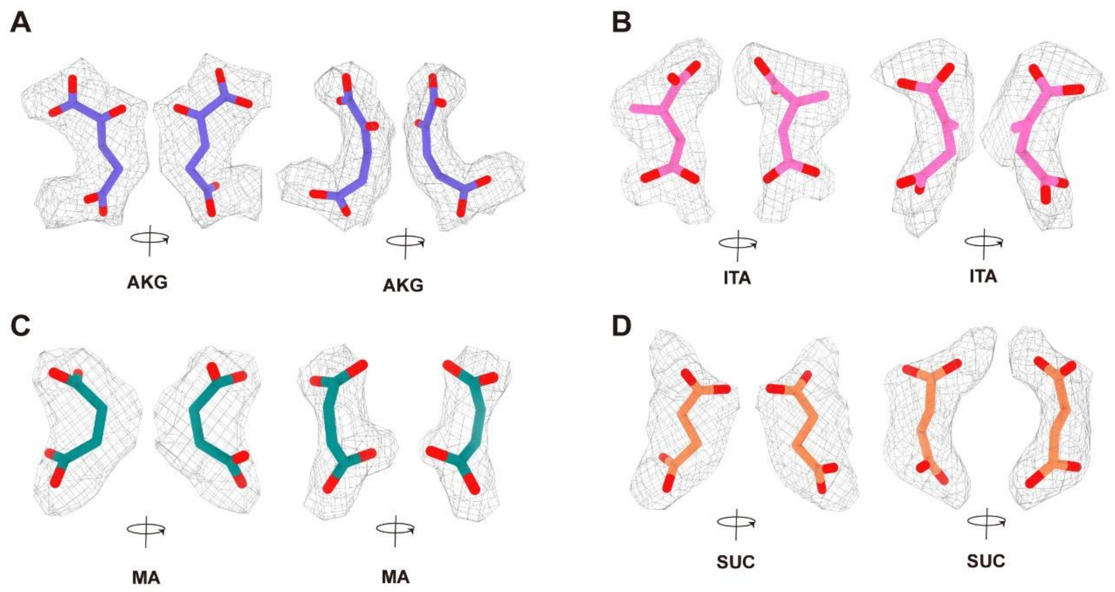

Figure. R1 | Multiple views of the ligand with corresponding EM density.

(A-D) Ligand densities were extracted from the locally refined maps and presented as mesh. All ligand-focused density maps are displayed at a contour level of 0.3.

2. The MD simulation trajectories should be deposited in the corresponding database as it has become a broadly followed practice in the field.

Response: We thank the referee for the suggestion. All MD simulation trajectories have been deposited in a figshare public repository, following widely adopted practices in the field, and are available at https://doi.org/10.6084/m9.figshare.28946102.v2.

3. The deviation from DRY and NPxxY motif is very intriguing, and mutagenesis data clearly demonstrate that D/E to F substitution in OXGR1 is functionally critical. The authors may consider testing P to L substitution in the NPxxY motif as well using endocytosis as readout.

Response: We thank the referee for this excellent suggestion. The NPxxY motif is vital for Class A GPCR activation. Our new calcium assays revealed that L299^{7x50}P mutant abolished activation of OXGR1 by AKG, ITA, SUC and MA (**Figure. R2A-D,** now included in **revised Figure. 6B**). Further, immunofluorescence assays showed that while WT-OXGR1 internalizes upon AKG stimulation, the L299^{7x50}P mutant remains at the plasma membrane (**Figure. R2E**). These results underscore the functional importance of the unique NLxxY motif in OXGR1.

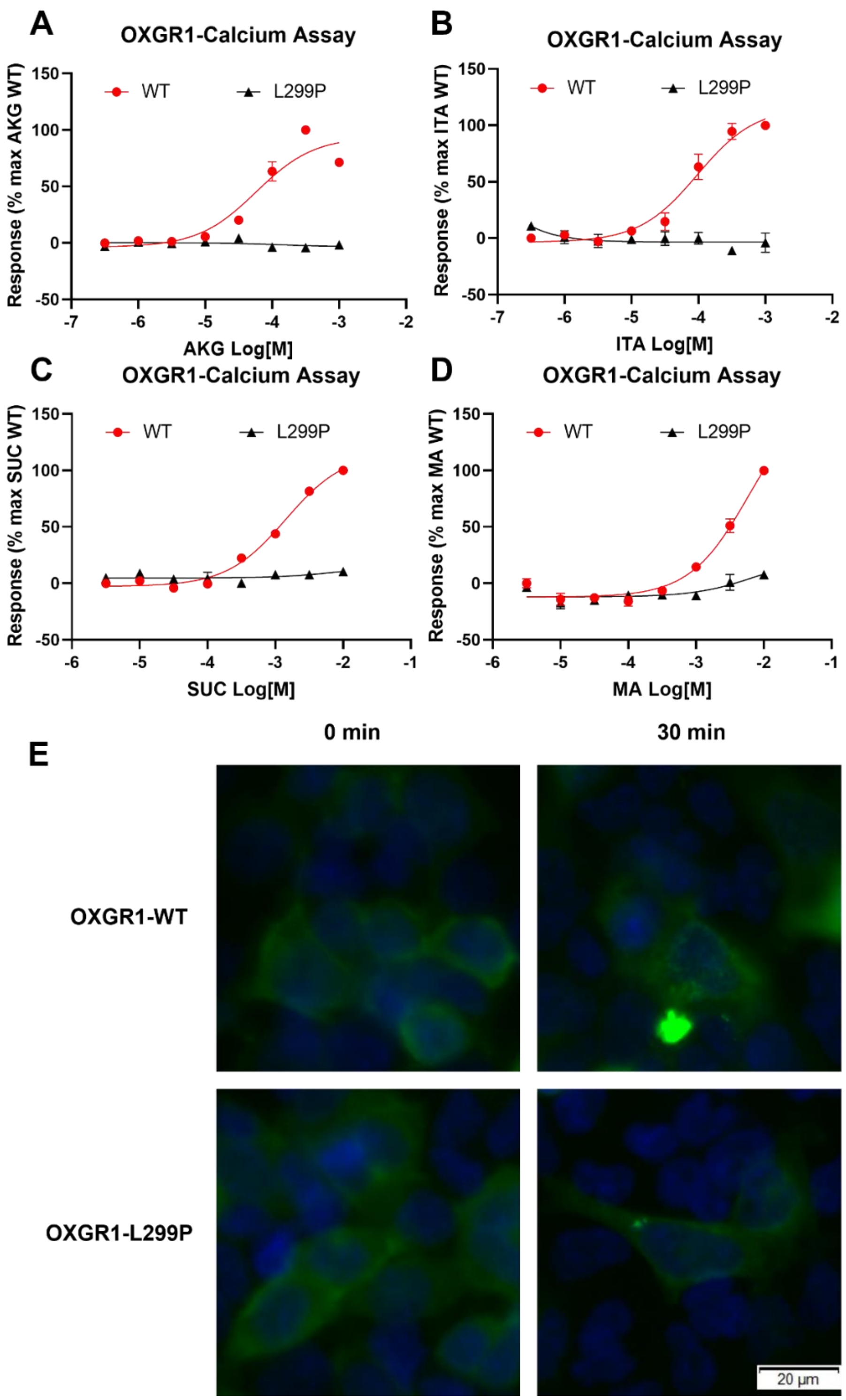

Figure. R2 | Effect of L299^{7x50}P mutant on activation and internalization of OXGR1.

(A-D) Effects of L299^{7x50}P mutant on the ligand AKG, ITA, SUC and MA induced calcium responses. Responses were normalized to 100% for wild-type. Data are mean ± S.E.M. from three independent experiments ($n = 3$), with three technical replicates performed for each experiment. **(E)** AKG induces internalization of OXGR1. HEK293 cells transiently transfected with HA-tagged OXGR1-WT or L299^{7x50}P mutant, were incubated with 300 μM AKG for 0 or 30 min at 37°C. OXGR1 were stained with anti-HA (green). Cell nuclei were

stained with Hoechst 33342 (blue). Scale bar, 20 μm.

4. Overall, the pharmacological assays are done reasonably well but, in some cases, where the response is observed only at the highest concentration e.g. metabolite screening or mutant validation, it could be more convincing to do a control with mock-transfected cells as well.

Response: We have followed this suggestion and conducted additional control experiments. As shown in **Figure R3**, while AKG, ITA, SUC, and MA show robust activity in OXGR1-transfected HEK293 cells, no activity was observed in mock-transfected cells. These results confirm the specificity of the ligands for OXGR1.

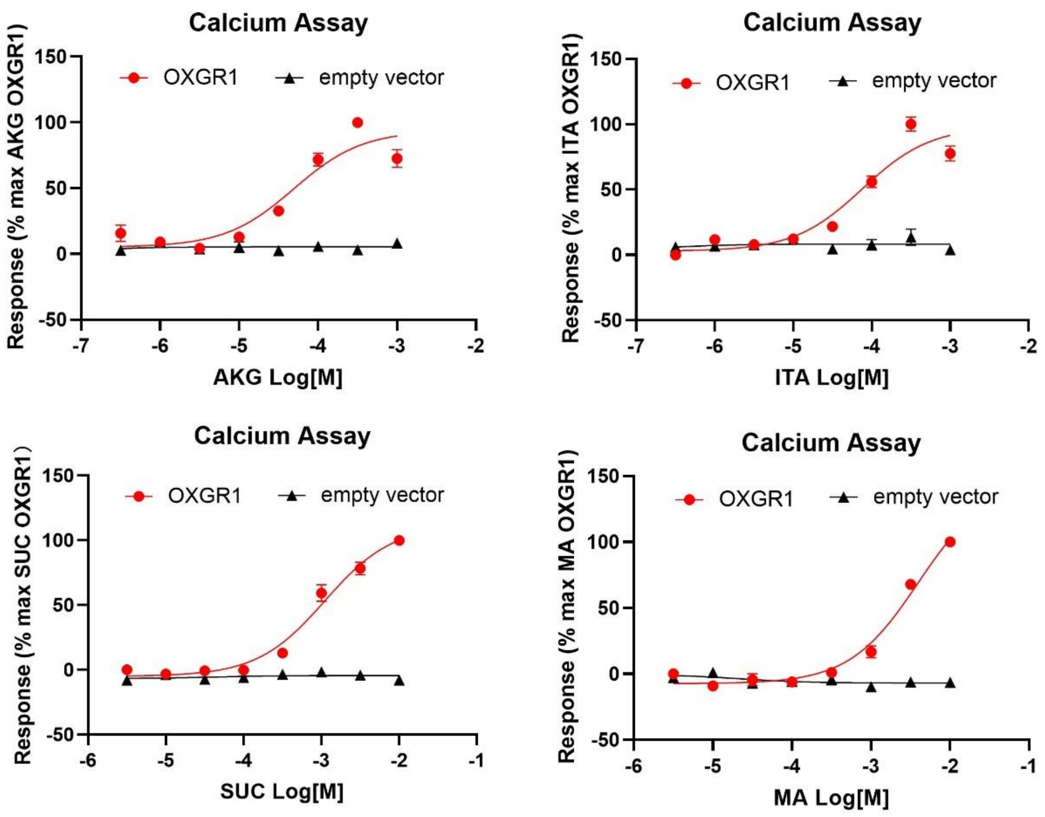

Figure. R3 | Activity of the compounds on OXGR1 using calcium assays.

Concentration-response curves of the four agonists in activating OXGR1 through the calcium assays. HEK293 cells were transfected with OXGR1 or empty vector by electroporation. Cells were then loaded with Fluo-4 AM and intracellular calcium changes after stimulation with different compounds were monitored. Responses were normalized to 100% for the maximal response of each compound on OXGR1. Data are mean ± S.E.M. from three independent experiments ($n = 3$), with three technical replicates performed for each experiment.

Arun K. Shukla, PhD

Referee #3:

Zhang et al have determined four cryo-EM structures of G protein-coupled OXGR1 bound to four different dicarboxylic acids SUC, MA, ITA and AKG (succinate, malate, itaconate and alpha-ketoglutarate). These metabolites play an important role in regulation of the metabolic state of cells, so the manuscript is an important step-change in our understanding of how these molecules promote signalling. The four dicarboxylic acids bind in a similar manner in the orthosteric binding pocket with important contributions being made by strong polar interactions with the positively charged residues R110, H114 and R288. A number of potential water molecules were also identified in the density maps. Mutation of these key residues and others involved in binding the ligands prevented or greatly reduced receptor activation. The relative contributions of the residues to binding were explored by molecular dynamics simulations to identify those elements most important for ligand potency. The authors then compare the structures with another GPCR that specifically binds succinate (SUCR1), and in a different manner to OXGR1. The authors elegantly show that they could alter the binding characteristics of SUCR1 through comparison with their OXGR1 structures, so that mutant SUCR1 gained binding of ITA that is normally unable to activate SUCR1. Finally, the authors perform mutagenesis to highlight residues that are involved in receptor activation in a manner analogous to other Class A GPCRs, despite having unique non-canonical motifs such as FRY rather E/DRY. Overall, the manuscript presents a comprehensive structure-function analysis of OXGR1.

The manuscript is very thorough, well-written and amply supported by many clear Figures. I have only a few queries and suggestions for clarifications that I have listed in the order they appear in the manuscript.

Response: We sincerely thank Referee #3 for the positive and insightful evaluation of our work and for recognizing the significance of our work. We also greatly appreciate the referee’s thoughtful comments and constructive suggestions, which have helped us further improve the clarity and rigor of the manuscript.

In the revised manuscript, we have expanded the Introduction, to more clearly describe the physiological context of OXGR1 signaling, including metabolite export mechanisms mediated by SLC transporters. To strengthen the mechanistic interpretation of receptor activation, we performed additional functional assays of the L299$^{7\times50}$P mutant, incorporating appropriate negative controls, which together confirm the essential role of the non-canonical NLxxY motif in OXGR1 activation. Structurally, we have standardized EM density visualizations, verified ligand protonation states at pH 7.4 using Epik, and consolidated the focused refinement statistics to emphasize the methodological rigor of our structural analysis.

Below, we respond to each specific comment point-by-point.

1. From the introduction, I am unclear about where signaling takes place. TCA cycle metabolites are usually thought to be found intracellularly, so does this mean that OXGR1 is on intracellular membranes? Or are the metabolites transported out of the cell, allowing them to signal to cells from the extracellular surface like many other GPCRs? A few sentences addressing this would be very helpful. I appreciate that this becomes obvious when reading the results section and perhaps lines 117-126 would be better in the introduction

Response: We appreciate the opportunity to clarify this. While TCA cycle metabolites are produced intracellularly, OXGR1 is a plasma-membrane receptor that senses extracellular ligands (He *et al*, 2004).

We have revised the Introduction (**Lines 95-113**) to explicitly describe how metabolites are exported via SLC transporters (e.g., SLC13A2/3/5 and SLC16A1/3) to reach the extracellular space, where they activate OXGR1 under various physiological and pathological conditions.

The revised text now explicitly as below:

"Intracellular TCA cycle metabolites can be exported to the extracellular space through specific solute carrier transporters (SLC) at the plasma membrane. These include Na^{+}/dicarboxylate cotransporters (SLC13A2/3/5) for the transport of AKG, ITA, SUC, and MA (Bergeron *et al*, 2013; Fan *et al*, 2025), as well as monocarboxylate transporters (SLC16A1/3) which mediate ITA export (Meng *et al*, 2025) and SUC release from muscle (Reddy *et al*, 2020). Consequently, the extracellular concentrations of these metabolites are dynamic. Under normal physiological conditions, ..."

2. Line 137-138. "Selective coupling to G_q-mediated signaling" implies OXGR1 couples only to G_q, but the authors tested only G_s and G_i and it is conceivable that other untested G proteins could couple. Please re-phrase to avoid introducing this misconception.

Response: We thank the referee for this important correction. We agree that "selective" might imply the exclusion of untested pathways. We have revised the text (**Lines 146-147**) to state that OXGR1:

"…primarily coupling to G_q-mediated signaling based on our comparative analysis with G_s and G_i pathways."

3. Please include a Figure where the four ligand orientations in the structures are compared after the structures have been aligned. In addition, four ligplot style Figures showing the residues involved in binding the respective ligands should be included to improve the clarity of the comparisons made.

Response: We thank the referee for this constructive suggestion. We have included a new supplementary figure (**revised Figure EV3A, and Figure. R4A below**) showing the superimposed structures of OXGR1 bound to all four ligands. This confirms their consistent horizontal orientation in the binding pocket. We also added Ligplot style diagrams for ITA, SUC, and MA (**revised Figure EV3B, and Figure. R4A below**) to highlight the shared and distinct interactions across the ligands.

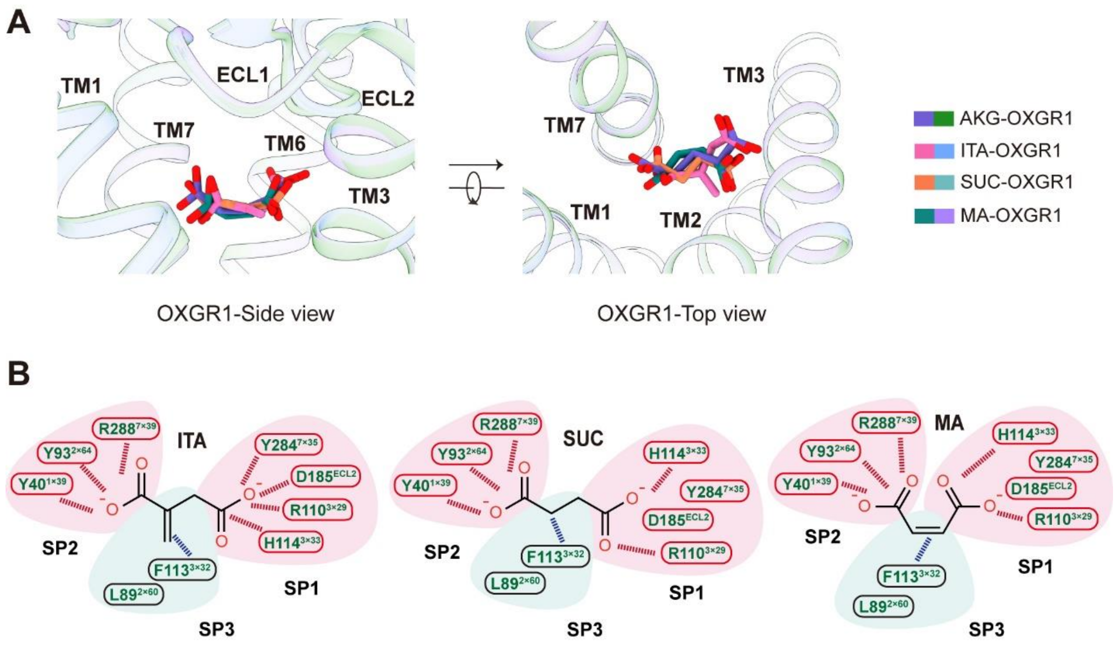

Figure. R4 (revised Figure EV3) | Structural comparison of ligand binding modes in OXGR1.

(A) Superimposed views of the four ligand-bound OXGR1 structures. These top and side views demonstrate that all four dicarboxylate ligands AKG, ITA, SUC, MA occupy a similar location within the orthosteric pocket and adopt a consistently horizontal orientation.

(B) The interaction ligplot style diagrams for the other three ligands (ITA, SUC, MA). Key polar interactions in the pink region are shown with red dashed lines and red circles, and hydrophobic interactions in the cyan region are shown with blue dashed lines and black circles. AKG/ITA engage in direct polar contacts with $D185^{45x52}$ and $Y284^{7x3}$, whereas these contacts are absent in SUC/MA.

4. Lines 209-211: I do not understand this sentence.

Response: We thank the referee for identifying this lack of clarity. We agree that the sentence was poorly formulated and potentially misleading and have removed it from the revised manuscript to ensure a more concise and logical flow.

5. Lines 244-247: There is 12 kcal/mol difference between the binding free energies for ITA and AKG and yet the EC_{50}s are virtually identical. How can this be? Please explain, especially given that the binding free energies for SUC and MA are virtually identical and so are their EC_{50}s.

Response: We appreciate the referee for the insightful question regarding the apparent discrepancy between the calculated binding free energies and the observed EC_{50} values. Binding free energy reflects the thermodynamic stability of the ligand-receptor complex, whereas EC_{50} is a functional parameter that depends on the efficiency with which ligand binding stabilizes an active receptor conformation and triggers downstream signaling.

Our MD simulations indicate that ITA and AKG both bind OXGR1 with sufficiently

favorable free energies to robustly populate an active receptor ensemble. Although ITA exhibits a substantially more favorable binding free energy than AKG, both ligands adopt highly similar binding poses (**Figures. 3D, E**) and engage key activation-associated residues, leading to comparable signaling efficiency. Under these conditions, additional binding stabilization does not further enhance functional potency, resulting in similar EC_{50} values.

In contrast, SUC and MA display markedly weaker binding free energies, which likely limit their ability to efficiently stabilize the active receptor conformation. Such a weak binding free energy may partially lead to disengaging from the binding pocket, which are expected to impair sustained receptor activation. The close similarity in both binding free energies and binding modes for SUC and MA is consistent with their nearly identical EC_{50}s.

Taken together, these results suggest that binding free energy differences explain the reduced potency of SUC and MA, whereas for ITA and AKG, binding strength exceeds the threshold required for efficient receptor activation, and functional potency is instead governed by shared binding poses and activation mechanisms.

We have also correspondingly revised **Lines 253-264** as:

"ITA exhibited the highest binding stability, with a binding free energy of -52.1 ± 0.9 kcal/mol (Fig. 4A), followed by AKG (-40.3 ± 3.3 kcal/mol). In contrast, SUC and MA displayed substantially weaker binding affinities (-31.8 ± 2.5 kcal/mol and -29.7 ± 6.0 kcal/mol, respectively) (Fig. 4A). Despite their difference in binding free energy, ITA and AKG exhibit comparable EC_{50} values, consistent with their highly similar binding poses and engagement of a shared set of activation-associated residues (Figs. 3D, E, and 4F, G), indicating that both ligands bind with sufficient strength to efficiently stabilize the active receptor conformation. In contrast, the markedly weaker binding free energies of SUC and MA likely limit efficient receptor activation, accounting for their reduced and nearly identical potencies. Further structural and predicted ranking of binding free energies revealed that residues $F113^{3x32}$, $D185^{45x52}$, and $Y284^{7x35}$ contribute differentially to ligand binding, depending on the specific chemical features of each metabolite (Fig. 4B-I)."

6. Line 254: I do not understand what is meant by 'pocket stabilization'

Response: We thank the referee for pointing this out. We have replaced the term "pocket stabilization" with a detailed description of the interaction. The revised text now reads:

"…In the AKG-bound structure, the α-keto carbonyl group engages in a σ-π interaction with $F113^{3x32}$, stabilizing the conformation of the binding pocket (Fig. 4C-E) …"

in **Lines 267-269** in our revised manuscript.

7. Line 380: delete 'succinate' and 'malate' as the abbreviations have been introduced before

Response: We have removed the redundant terms in the revised manuscript (**Line 396**) to ensure consistent use of the abbreviations "SUC" and "MA" throughout the text.

8. Figure 1: In panel B, the structures are given of the protonated forms yet the pKas of the acidic groups are between about 2-5, and therefore would be expected to be unprotonated at pH 7.4 (buffer used for purification). The authors also mention that they anticipate the acids would be unprotonated given the proximity of positively charged residues in the structures. Please could you therefore draw the structures as being unprotonated and show the delocalized electron structures. It would be helpful in the text if the pKas of the acidic groups are mentioned and state they are unprotonated.

Response: We sincerely thank the referee for the attention. The protonation states of the acidic groups were evaluated using Epik, and the calculated pKa values are as follows (acidic groups from left to right in **Figure. 1B**): AKG, 3.46 and 4.28; ITA, 4.96 and 4.84; SUC, both 5.17; and MA, both 6.11. At pH 7.4, all acidic groups are therefore expected to be fully deprotonated. We have mentioned such values in figure legends and shown the delocalized electron structures in **revised Figures.1B** of our revised manuscript.

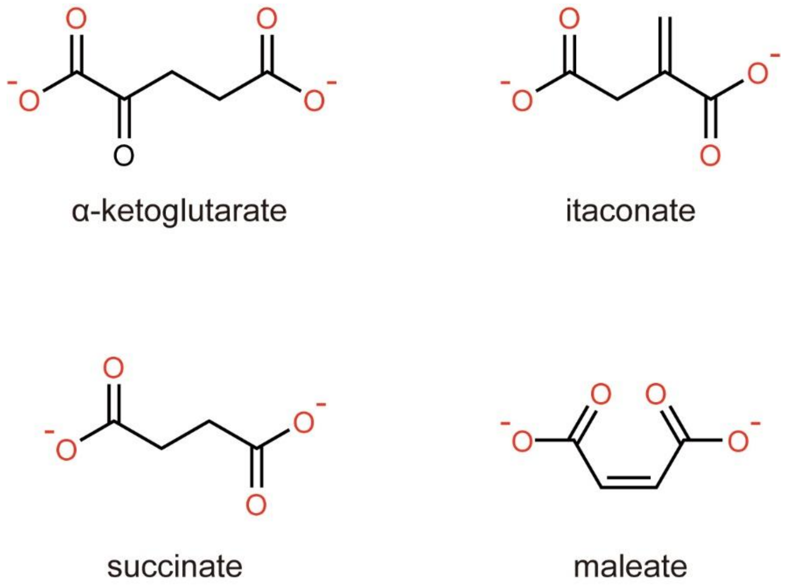

Figure. R5 | Deprotonated structures of AKG, ITA, SUC and MA.

9. Figure 1: In panel D it seems strange that all the curves stop at 100% and the curves for SUC and MA have not plateaued. The dicarboxylic acids have high solubility in water so I would have thought it would have been possible to use higher concentrations. In addition, what is the size of the assay window in this experiment? This should be much larger than the window of the recorded response, but if it is limited to 100% then it could explain why all the curves finish at the same level.

Response: We sincerely thank the referee for this insightful comment. Following your suggestion, we extended the concentration range for all four ligands in our calcium flux assays. The revised data, now normalized to the maximal response of AKG (defined as 100%), reveal that SUC and MA reached higher plateaus at approximately 150% relative to AKG (**Figure R6 and revised Figure 1D**).

Regarding the titration of AKG and ITA, we observed a decrease in signal at concentrations

exceeding our reported range. This "bell-shaped" response curve is likely attributable to non-specific effects or ligand-induced cellular toxicity at extremely high concentrations, a phenomenon previously noted for certain dicarboxylates. Consequently, we have reported the curves up to the concentration that elicits the maximal physiologically relevant response (**Figure R6 and revised Figure 1D**). The previous normalization method (capping all ligands at 100%) has been updated to this unified normalization against AKG to more accurately reflect the relative efficacies of the metabolites. These new data provide more accurate activity profiles of four ligands at OXGR1.

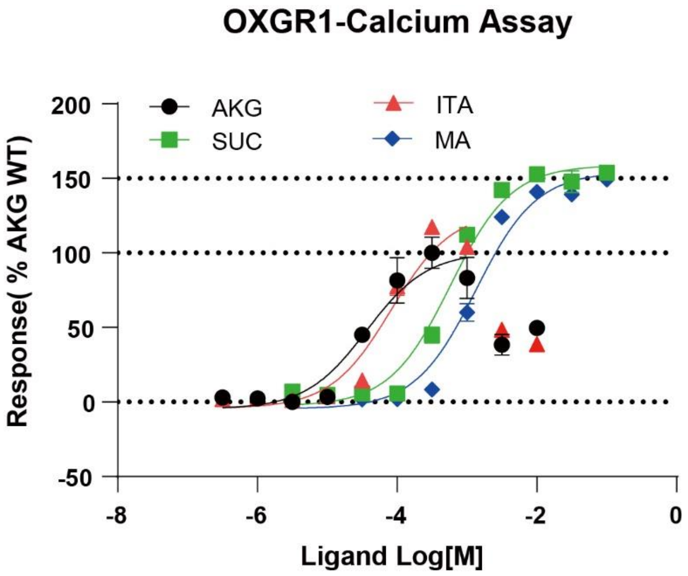

Figure. R6 | Comparative efficacy of TCA metabolites on OXGR1.

Concentration-response curves of four agonists (AKG, ITA, SUC, and MA) in activating OXGR1 were determined via calcium flux assays in HEK293 cells. Intracellular calcium transients were monitored using the fluorescent indicator Fluo-4 AM. To facilitate comparison of relative efficacy, all responses were normalized to the maximal response elicited by AKG (100%). Data represent the mean ± S.E.M. from three independent experiments ($n = 3$), with three technical replicates performed for each experiment.

10. Figure 1, line 10 and panel C: Please do not use the term NA for 'not active'. In English it is a standard abbreviation for 'not applicable'. I would just delete this and put outlines of boxes for white areas that showed no binding. In addition, just write 'Not tested' in the area where particular combinations were not tried and perhaps colour this solid grey for clarity.

Response: We thank the referee for this thoughtful suggestion regarding the clarity of our data presentation. We agree that "N.A." is ambiguous in this context. Accordingly, we have updated **Figure 1C** (also shown as **Figure R7**) with the following refinements:

1, The abbreviation “N.A.” has been removed to avoid confusion with "not applicable."

2, Areas representing no detectable activity are now indicated with a white background, consistent with the pEC_{50} color scale.

3, Combinations that were not tested are now filled with solid grey and explicitly labeled as "Not tested".

These adjustments ensure a clearer distinction between lack of activity and lack of data, facilitating a more accurate interpretation of the receptor screening results.

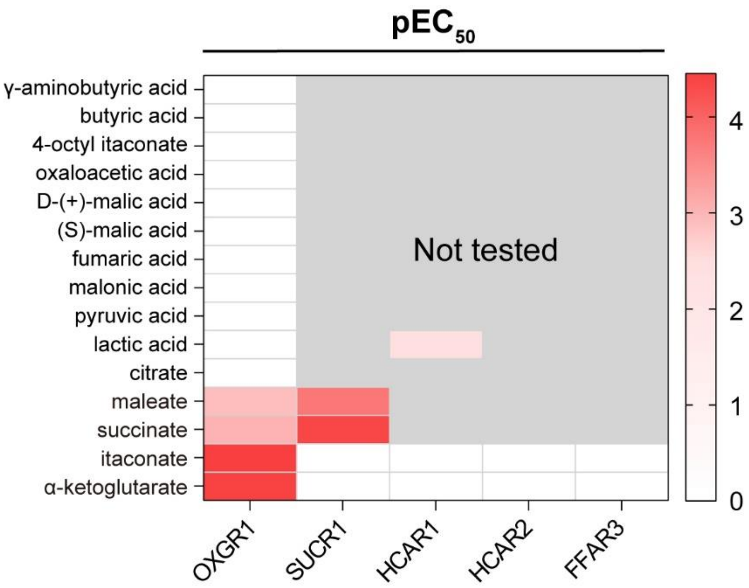

Figure. R7 | Efficacy profile of TCA metabolites and derivatives across carboxylic acid-sensing receptors.

Heat map characterization of the activities of AKG, ITA, and related metabolites on OXGR1 and other carboxylic acid-sensing receptors, as determined by calcium flux assays. The heat map is colored based on pEC_{50} values. White cells indicate no detectable activation, while solid grey cells labeled "Not tested" represent combinations that were not included in the assay. Data represent the mean ± S.E.M. from three independent experiments ($n = 3$), with three technical replicates performed for each experiment.

11. In all panels containing plotted data, please state the number of technical replicates per experiment after stating the number of independent experiments e.g. 'three independent experiments were performed in duplicate'. Please also give a link to a table of the numerical data that was plotted, along with EC_{50}s where relevant and their respective errors.

Response: We thank the referee for this suggestion regarding data transparency. We have updated the figure legends for all panels containing plotted data to explicitly state both the number of independent experiments and technical replicates. Specifically, the legends now state: "Data are presented as mean ± S.E.M. of three independent experiments ($n = 3$), with three technical replicates performed for each experiment".

Furthermore, we have provided the corresponding numerical data, including EC_{50} values and their respective errors, in **Appendix Tables S1, S4 and S5**. These tables are linked in the

revised manuscript to facilitate further data assessment.

12. Figure 4, panel D: Please define what distance is being measured here e.g. is it between specific atoms (which?) or a centre of mass (COM)?

Response: We thank the referee for raising this point and allowing us to clarify the structural measurements. The distances depicted in **Figure 4D** represent the minimal distance between any non-hydrogen atom of the ligand and any non-hydrogen atom of the specified residue. We have updated the figure legend to explicitly state this definition and avoid any ambiguity regarding the measurement method.

13. Figure 4, line 78-79. It is difficult to distinguish between the blue and red lines in the Figure.

Response: We thank the referee for identifying these visualization issues. To improve clarity, we have explicitly defined these line styles in the revised figure legend. Furthermore, we have enhanced the color contrast in the Figure to ensure all elements are easily distinguishable, as shown in **Figure. R8**.

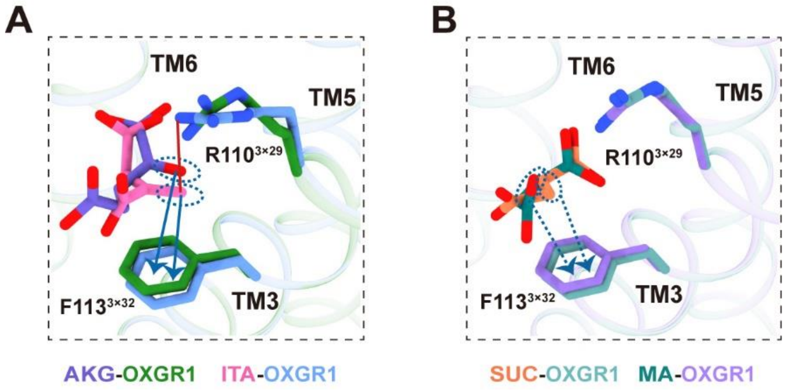

Figure. R8 | Comparison of AKG/ITA with SUC/MA interaction with F113^{3x32} in OXGR1 pocket.

The electrostatic interaction between the double bond of ITA and R110^{3x29}, represented by a solid red line (**A**). The σ-π/π-π stacking interaction between AKG/ITA and F113^{3x32}, represented by a solid blue line with dashed blue circle (**A**). The C-H-π stacking interaction between SUC/MA and F113^{3x32}, represented by a dashed blue line with dashed blue circle (**B**). The blue solid lines represent more stable interactions compared to the blue dashed lines.

14, Lines 80-82, I cannot see this in the Figure.

We apologize for the lack of clarity in the previous version. To make these observations more apparent, we have updated **Figure 4D** (shown as **Figure R9**) by adding vertical dashed lines that point directly to the peak interatomic distances on the X-axis. As shown in the revised panel:

Top Panel (R110^{3x29}): All four ligands (AKG, ITA, SUC, and MA) maintain a similar minimal distance, reflecting a conserved electrostatic interaction.

Bottom Panel (F113^{3x32}): There is a clear divergence. AKG and ITA maintain a distinct and

consistent distance from F113^{3x32} that differs significantly from the distances observed for SUC and MA. These visual aids, combined with updated color contrast, now clearly illustrate the distinct interaction profiles of these metabolites.

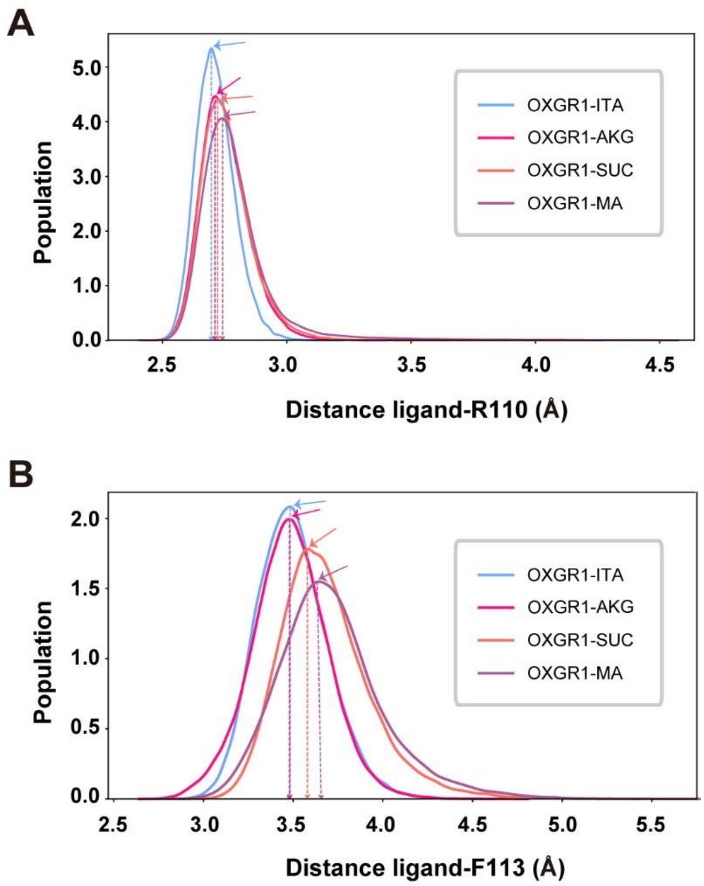

Figure. R9 | Interatomic distance distributions reveal distinct interaction profiles.

Histograms showing the minimal distances between non-hydrogen atoms of each ligand and residues R110^{3x29} (top) and F113^{3x32} (bottom) during MD simulations. Vertical dashed lines indicate the peak distribution values on the X-axis for each ligand. Note that AKG and ITA exhibit a different distance profile relative to F113^{3x32} compared to SUC and MA.

15. Figures EV2 & EV3: In the local resolution maps (E, L, G, N), please choose appropriate scales for each panel so that everything is not all blue e.g. between 2 Å to 4 Å.

Response: We thank the referee for this constructive suggestion regarding the visualization of our cryo-EM maps. In the revised **Appendix Figures S2 and S3**, we have adjusted the local resolution color scales to a range of 2-4 Å (**Figure. R10**). This optimized scaling significantly enhances contrast, allowing for a more detailed assessment of the local resolution variations.

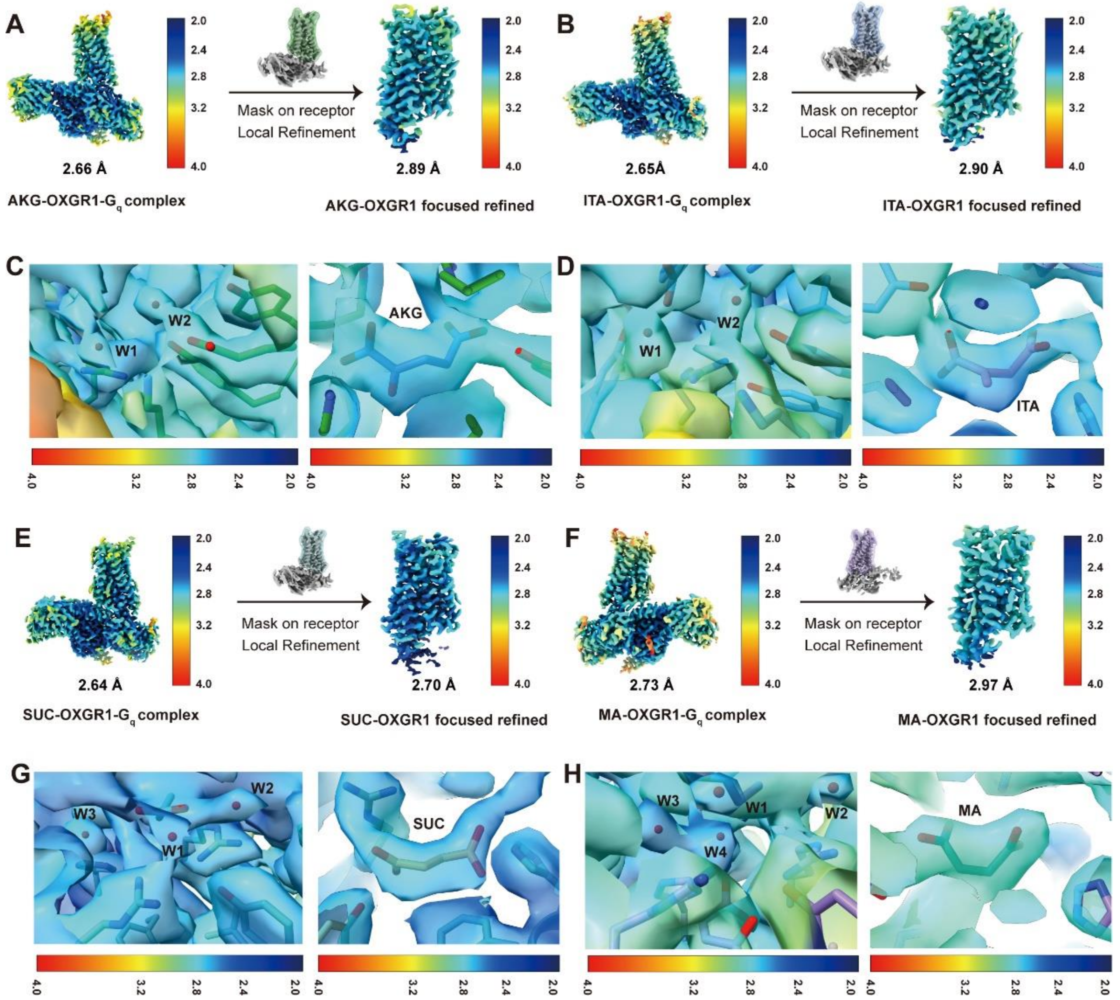

Figure. R10 | Global and local resolution maps of the OXGR1-G_q complexes in different ligand-bound states.

The cryo-EM density maps for the receptor, ligands, and coordinated water molecules are colored according to local resolution.

16. Figure EV9. What do the blank circles mean?

Response: We thank the referee for the opportunity to clarify our data visualization. In revised **Appendix Figure S6 (initial Figure EV9)**, the blank circles represent specific residues that were included in the analysis but do not form direct interactions with the ligands in the given structure. We have updated the figure legend to explicitly define this representation and ensure clarity for the reader.

17. Tables S1 and S2. Extra columns have been included for the receptor alone after focused refinement. I am not sure this is necessary and may be confusing. The extra columns imply that they are different structures when in fact the receptor structures are absolutely identical (RMSD 0.000). Please make it clear in the Methods and these tables that the receptor models are the same. Perhaps only have one column and put the important numbers (resolution, number of water molecules) in parentheses for the focused refined model and give a footnote to the table? In the results section of the main text, it would also be worth mentioning the improvement in density that you see on focused refinement of the receptors (not nearly

enough people are doing receptor focused refinements).

Response: We sincerely thank the referee for this insightful and constructive suggestion regarding the presentation of our refinement statistics. We have fully implemented these changes to clarify that the models are identical while highlighting the methodological benefit of focused refinement.

Specifically:

Appendix Tables S2 and S3: We have consolidated the columns. Statistics from the receptor-focused refinement (e.g., local resolution and number of modeled water molecules) are now provided in parentheses alongside the global values. A detailed footnote has been added to explain this format.

Methods Section: We have updated the image processing description to explicitly state that local refinement was performed to enhance the map quality of the receptor region, reaching resolutions up to 2.89 Å.

Results Section: In accordance with the referee's suggestion, we now explicitly mention the improvement in map quality: "To enhance the visualization of ligand densities and side-chain conformations, we performed focused refinement on the receptor, achieving local resolutions of 2.89 Å, 2.90 Å, 2.70 Å, and 2.97 Å for the respective complexes (**Appendix Tables S2 and S3**)."

We believe these revisions eliminate potential confusion and correctly emphasize the technical rigor of our structural analysis.

Appendix Table S2 for example:

Appendix Table S2| Cryo-EM data collection, refinement, and validation statistics. Statistics are provided for the OXGR1-AKG-G_q and OXGR1-ITA-G_q complexes, as well as the corresponding receptor-focused refinements (related to **Figure 2 and Appendix Figure S2**)

**Data Collection**		
**Protein**	**AKG-OXGR-G_q complex (OXGR1 focused refined)**	**ITA-OXGR1-G_q complex (OXGR1 focused refined)**
**Voltage (kV)**	300	300
**Detector**	K3	K3
**Pixel size (Å)**	0.82	0.82
**Defocus range (µm)**	1.0-2.0	1.0-2.0
**Electron dose**	50	50
**3D Reconstruction**		
**Final Particle**	405,687	405,209
**Symmetry**	C1	C1
**Overall resolution**	2.66 (2.89)	2.65 (2.90)
**Model refinement**		
**Model composition**		
**Chains**	7 (3)	7 (3)
**Water**	(2)	(2)
**Ligands**	1	1
**Non-hydrogen**	9,870 (2,309)	9,840 (2,309)
**Protein residues**	1,264 (286)	1,264 (286)
**Bonds (RMSD)**		
**Length (Å)**	0.003	0.003
**Angles (°)**	0.547 (0.561)	0.596 (0.616)
**Ramachandran plot**		
**Outliers**	0.00	0.00
**Allowed**	1.53 (1.76)	1.77 (2.11)
**Favored**	98.47 (98.24)	98.23 (97.89)
**Rotamer outlier**	0.37 (0.38)	0.19 (0.38)
**MolProbity score**	1.18 (1.40)	1.31 (1.22)
**Clash score**	3.93 (7.25)	5.64 (4.06)

Note: Data in parentheses represent the results of local and focused refinements performed on the OXGR1 receptor region of each complex. As detailed in the Methods and Results, this focused approach significantly improved the visualization of ligand densities, side-chain conformations, and coordinated water molecules within the orthosteric pocket.

Typos

Line 156: deletion “portion”

We have deleted “portion” in **Line 165** in our manuscript.

Line 166: insert “was' after 'arrangement”

We have added “was” in **Line 175** in our manuscript.

Line 195: change to “the four metabolites AKG, ITA...”

We have changed this in **Line 204** in our manuscript.

Line 215: do not use possessives (ligands’)

We have fixed this in **Lines 224-225** in our manuscript.

Line 238: change to “...different ligand potencies”.

We have changed this in **Line 247** in our manuscript.

Referee #4:

α-ketoglutarate (AKG) and itaconate (ITA) are important metabolites and OXGR1 plays critical roles in metabolism regulation by sensing AKG and ITA. This manuscript reported the Cryo-EM structures of active state OXGR1 bound with AKG and ITA, as well as the related but weaker binding metabolites succinate (SUC) and maleate (MA) at the 3.0Å higher resolutions. The ligands and the binding environment are clearly identified and are consistent with the results of molecular docking. Systematic mutagenesis and activity assay proved the roles of critical residues in ligand binding and signal transduction. The experimental data are solid and the manuscript is well written. This manuscript provides deep insights into specific ligand recognition and G protein coupling mechanism and may have contribution to future drug design targeted to OXGR1.

Only a concern should be addressed: the density map in Figure 2, Figure EV4 and EV5 look different, especially in Figure EV5, although all of the contour levels are 0.3.

Response: We sincerely thank Referee #4 for the positive assessment and careful evaluation of our work. We appreciate the referee’s attention to the consistency of our map visualizations.

The perceived differences in the density maps in the initial version were due to the use of different volume segmentation methods. In **initial Figure EV5**, the map was visualized within a segmented volume that included both the ligand and adjacent residues (Y40^{1x39}, H114^{3x33}, and R288^{7x39}), which resulted in “connected” density, as shown in **Figure. R11**. In contrast, **initial Figures 2 and EV4** utilized a more focused contour surface centered strictly on the ligand, providing clearer isolation of its density.

To ensure uniformity, we have now standardized the visualization across all figures using the ligand-focused isolation method. This update is reflected in the **revised Figure EV1** (also

shown in **Figure. R12** below). This adjustment ensures a consistent and accurate representation of the ligand fit within the EM density throughout the manuscript without altering the underlying structural models.

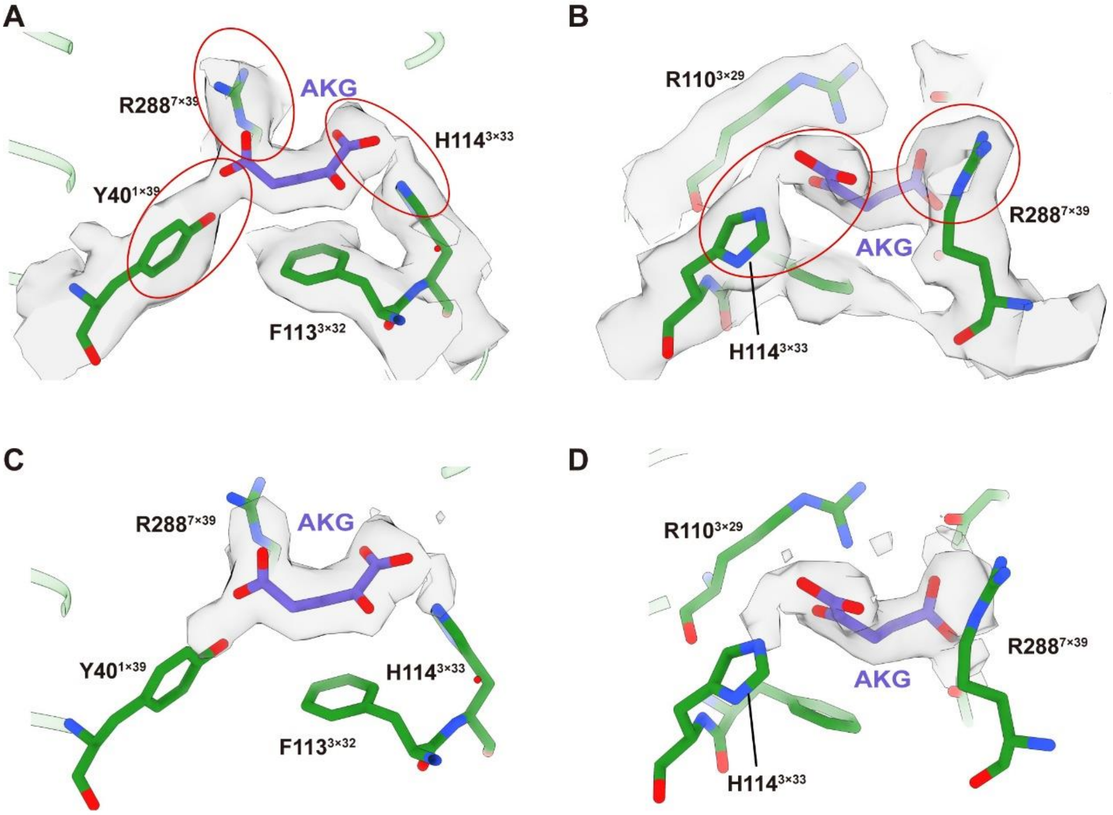

Figure. R11 | Connectivity of the ligand density to OXGR1 binding pocket using AKG as an example.

(A-B) Close-up view of the ligand-protein interface, with the map contoured at a level of 0.3. Red circles mark areas of continuous EM density connecting AKG to surrounding residues Y40^{1x39}, H114^{3x33}, R288^{7x39}; (**C-D**) At the same view as shown above, the EM density map of the ligand AKG, displayed within a segmented volume. The ligand density appears continuous with that of nearby residues.

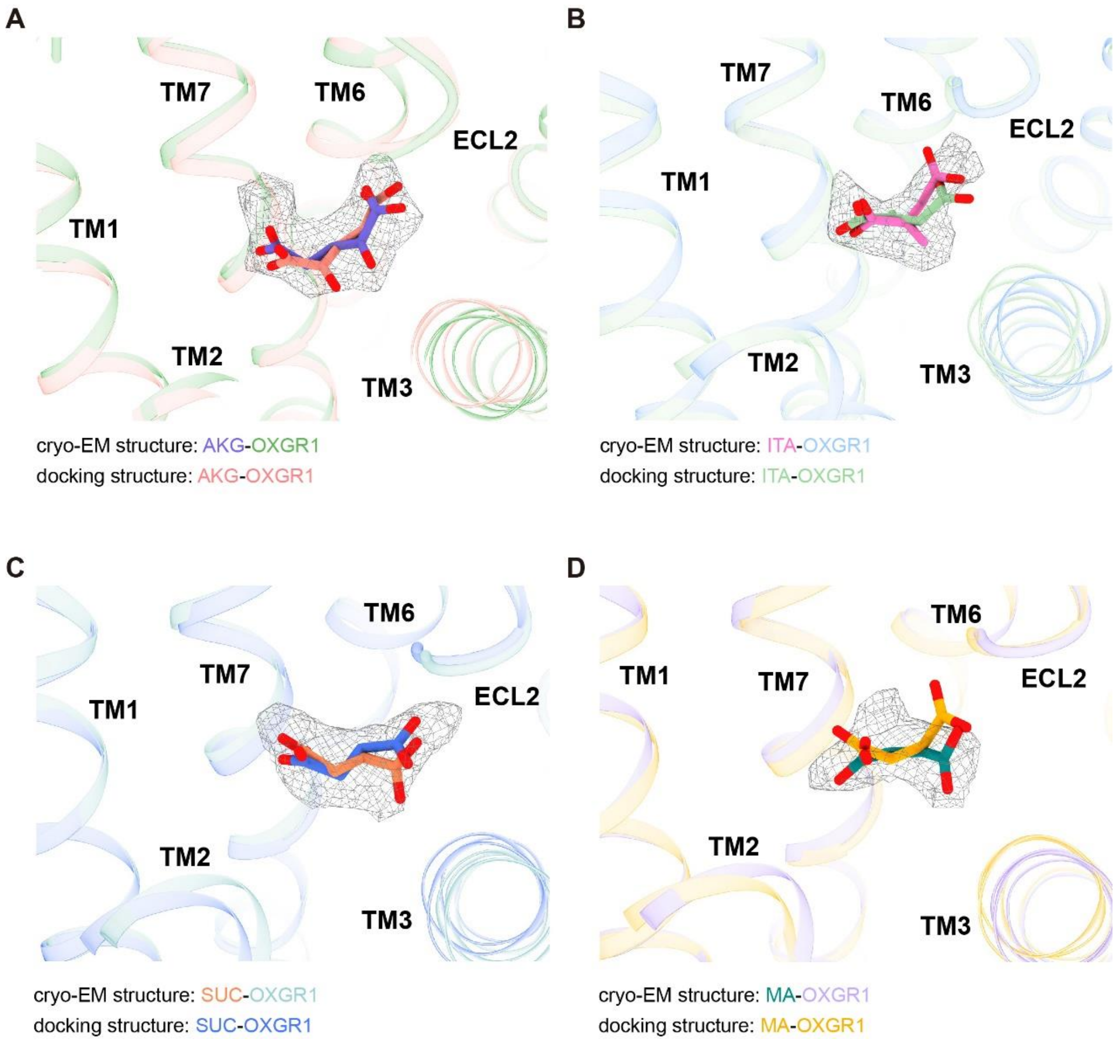

Figure. R12 (revised Figure EV1) | Structural validation of four ligand-binding modes in OXGR1 by molecular docking and cryo-EM density maps.

Comparison of AKG **(A)**, ITA **(B)**, SUC **(C)**, and MA **(D)** binding postures in molecular docking and structural modeling of OXGR1. Each ligand is aligned with the corresponding cryo-EM density represented in mesh, validating the proposed binding conformations. The densities of respective ligands have been extracted from their local structures of receptors and shown in surface presentation. All ligand-focused density maps are shown at contour level of 0.3, excluding connected density from surrounding residues, and the local resolutions of AKG/ITA/SUC/MA-bound OXGR1 are 2.89 Å, 2.90 Å, 2.70 Å, 2.97 Å, respectively.

Reference

Bergeron MJ, Clémençon B, Hediger MA, Markovich D (2013) SLC13 family of Na+-coupled di- and tri-carboxylate/sulfate transporters. *Molecular Aspects of Medicine* 34: 299-312

Fan Y, Dan W, Wang Y, Ma Z, Jian Y, Liu T, Li M, Wang Z, Wei Y, Liu B et al (2025) Itaconate transporter SLC13A3 confers immunotherapy resistance via alkylation-mediated stabilization of PD-L1. *Cell Metabolism* 37: 514-526.e515

He W, Miao FJP, Lin DCH, Schwandner RT, Wang Z, Gao J, Chen J-L, Tian H, Ling L (2004) Citric acid cycle intermediates as ligands for orphan G-protein-coupled receptors. *Nature* 429: 188-193

Hochachka PW, Dressendorfer RH (1976) Succinate accumulation in man during exercise. *Eur J Appl Physiol Occup Physiol* 35: 235-242

Hochachka PW, Owen TG, Allen JF, Whittow GC (1975) Multiple end products of anaerobiosis in diving vertebrates. *Comparative Biochemistry and Physiology Part B: Comparative Biochemistry* 50: 17-22

Kushnir MM, Komaromy-Hiller G, Shushan B, Urry FM, Roberts WL (2001) Analysis of dicarboxylic acids by tandem mass spectrometry. High-throughput quantitative measurement of methylmalonic acid in serum, plasma, and urine. *Clin Chem* 47: 1993-2002

Meng Q, Li C, Cai Y, Chen Y, Chen X, Wang X, Zhang B, Zhang Y, Liu F, Chen M (2025) Itaconate transport across the plasma membrane and Salmonella-containing vacuole via MCT1/4 modulates macrophage antibacterial activity. *Nature Communications* 16: 10551

Mills EL, Ryan DG, Prag HA, Dikovskaya D, Menon D, Zaslona Z, Jedrychowski MP, Costa ASH, Higgins M, Hams E et al (2018) Itaconate is an anti-inflammatory metabolite that activates Nrf2 via alkylation of KEAP1. *Nature* 556: 113-117

Reddy A, Bozi LHM, Yaghi OK, Mills EL, Xiao H, Nicholson HE, Paschini M, Paulo JA, Garrity R, Laznik-Bogoslavski D et al (2020) pH-Gated Succinate Secretion Regulates Muscle Remodeling in Response to Exercise. *Cell* 183: 62-75.e17

Yuan Y, Xu P, Jiang Q, Cai X, Wang T, Peng W, Sun J, Zhu C, Zhang C, Yue D et al (2020) Exercise-induced α-ketoglutaric acid stimulates muscle hypertrophy and fat loss through OXGR1-dependent adrenal activation. *The EMBO Journal* 39: e103304

1st Revision - Editorial Decision 11th Mar 2026

Dear Dr. Liu,

Thank you for submitting a revised version of your manuscript. Your study has now been seen by two of the original referees, who find that their previous concerns have been addressed and now recommend publication of the manuscript. There remain only a few mainly editorial points that have to be addressed before I can extend formal acceptance of the manuscript:

- Please rename the Conflict of Interest section into "Disclosure and Competing Interests Statement", in accordance with our updated Guide to Authors (https://link.springer.com/partners/embo-press/editorial-policies#Competing%20interest%20disclosures)

- AFFILIATIONS: affiliation #4 might be a company - employment in a biotech company should be stated in DCIS

- As we are switching from a free-text author contribution statement towards a more formal statement based on Contributor Role Taxonomy (CRediT) terms, please remove the present Author Contribution section and instead specify each author's contribution(s) directly in the Author Information page of our submission system during upload of the final manuscript. See https://casrai.org/credit/ for more information.

Please convert the APPENDIX FILE WITH ToC into PDF format

- Please provide suggestions for a short 'blurb' text prefacing and summing up the conceptual aspect of the study in two sentences (max. 250 characters), followed by 3-5 one-sentence 'bullet points' with brief factual statements of key results of the paper; they will form the basis of an editor-written 'Synopsis' accompanying the online version of the article. Please also provide an altered synopsis image, making sure that the aspect ratio conforms to our website's format - it should be exactly 550 pixels wide and between 300-600 pixels high.

- The manuscript sections should be in the following order: Title page - Abstract & Keywords - Introduction - Results - Discussion - Methods - Data Availability - Acknowledgments - Disclosure Statement & Competing Interests - References - Figure Legends - (Main Tables with legends if applicable) - Expanded View Figure Legends.

- Materials and Methods should be Methods

- Please note that the specific URLs for EMD 63580, EMD 64590, EMD 63581, EMD 64589, EMD 63583, EMD 63582, EMD 64587, EMD 64588, 9M1R, 9UXQ, 9M1S, 9UXP, 9M1U, 9UXN, 9M1T, and 9UXO. datasets are not provided in the data availability statement.

- Please note that the exact p values are not provided in the legends of figures 4E, 5G

Please let me know if you have any questions regarding any of these points. You can use the link below to upload the revised files.
Thank you again for giving us the chance to consider your manuscript for The EMBO Journal. I look forward to receiving the final version.

With best regards,

Cornelius Schneider

Cornelius Schneider, PhD
Editor | The EMBO Journal
c.schneider@embojournal.org

Please refer to our figure preparation guideline in order to ensure proper formatting and readability in print as well as on screen:

https://link.springer.com/journal/44318/submission-guidelines#cms-Figure-and-data-presentation

Use the link below to submit your revision:

https://emboj.msubmit.net/cgi-bin/main.plex

Referee #2:

The authors have addressed all the points raised during the original review, and I recommend publication of this manuscript.
Arun K. Shukla, PhD

Referee #3:

The authors have considered carefully the comments and have made the necessary changes to the manuscript. I have no further comments.

All editorial and formatting issues were resolved by the authors.

2nd Revision - Editorial Decision 5th May 2026

Dear Dr. Liu,

I am pleased to inform you that your manuscript has been accepted for publication in the EMBO Journal.

Your manuscript will be processed for publication by EMBO Press. It will be copy edited and you will receive page proofs prior to publication. Please note that you will be contacted by Springer Nature Author Services to complete licensing and payment information.

You may qualify for financial assistance for your publication charges - either via a Springer Nature fully open access agreement or an EMBO initiative. Check your eligibility: https://link.springer.com/journal/44318/how-to-publish-with-us

Should you be planning a Press Release on your article, please get in contact with embo_production@springernature.com as early as possible in order to coordinate publication and release dates.

If you have any questions, please do not hesitate to contact the Editorial Office. Thank you for your contribution to The EMBO Journal.

Yours sincerely,

Cornelius Schneider, PhD
Editor
The EMBO Journal
c.schneider@embojournal.org

*****
Please note that it is The EMBO Journal policy for the transcript of the editorial process (containing referee reports and your response letters) to be published as an online supplement to each paper. If you should prefer removal of any referee-only figures included in the point-by-point response(s), e.g. because they may still be used for future publication or because they have been reproduced from published work by others, please do let us know immediately via response email.
More information is available here: https://link.springer.com/partners/embo-press/editorial-policies#Peer%20review

** Click here to be directed to your login page: https://emboj.msubmit.net

EMBO Press Author Checklist

Corresponding Author Name: Heng Liu, H. Eric Xu, Xin Xie
Journal Submitted to: The EMBO Journal
Manuscript Number: EMBOJ-2025-122560

USEFUL LINKS FOR COMPLETING THIS FORM
The EMBO Journal - Author Guidelines
EMBO Reports - Author Guidelines
Molecular Systems Biology - Author Guidelines
EMBO Molecular Medicine - Author Guidelines

Reporting Checklist for Life Science Articles (updated January
This checklist is adapted from Materials Design Analysis Reporting (MDAR) Checklist for Authors. MDAR establishes a minimum set of requirements in transparent reporting in the life sciences (see Statement of Task: 10.31222/osf.io/9sm4x). Please follow the journal's guidelines in preparing your
Please note that a copy of this checklist will be published alongside your article.

Abridged guidelines for figures
1. Data
The data shown in figures should satisfy the following conditions:
- the data were obtained and processed according to the field's best practice and are presented to reflect the results of the experiments in an accurate and unbiased manner.
- ideally, figure panels should include only measurements that are directly comparable to each other and obtained with the same assay.
- plots include clearly labeled error bars for independent experiments and sample sizes. Unless justified, error bars should not be shown for technical
- if n<5, the individual data points from each experiment should be plotted. Any statistical test employed should be justified.
- Source Data should be included to report the data underlying figures according to the guidelines set out in the authorship guidelines on Data

2. Captions
Each figure caption should contain the following information, for each panel where they are relevant:
- a specification of the experimental system investigated (eg cell line, species name).
- the assay(s) and method(s) used to carry out the reported observations and measurements.
- an explicit mention of the biological and chemical entity(ies) that are being measured.
- an explicit mention of the biological and chemical entity(ies) that are altered/varied/perturbed in a controlled manner.
- the exact sample size (n) for each experimental group/condition, given as a number, not a range;
- a description of the sample collection allowing the reader to understand whether the samples represent technical or biological replicates (including how many animals, litters, cultures, etc.).
- a statement of how many times the experiment shown was independently replicated in the laboratory.
- definitions of statistical methods and measures:
  - common tests, such as t-test (please specify whether paired vs. unpaired), simple χ2 tests, Wilcoxon and Mann-Whitney tests, can be unambiguously identified by name only, but more complex techniques should be described in the methods section;
  - are tests one-sided or two-sided?
  - are there adjustments for multiple comparisons?
  - exact statistical test results, e.g., P values = x but not P values < x;
  - definition of 'center values' as median or average;
  - definition of error bars as s.d. or s.e.m.

Please complete ALL of the questions below.
Select "Not Applicable" only when the requested information is not relevant for your study.

Materials

Newly Created Materials	Information included in the manuscript?	In which section is the information available? (Reagents and Tools Table, Materials and Methods, Figures, Data Availability Section)
New materials and reagents need to be available; do any restrictions apply?	Not Applicable	

Antibodies	Information included in the manuscript?	In which section is the information available? (Reagents and Tools Table, Materials and Methods, Figures, Data Availability Section)
For **antibodies** provide the following information:<br>- Commercial antibodies: RRID (if possible) or supplier name, catalogue number and or/clone number<br>- Non-commercial: RRID or citation	Yes	Reagents and Tools Table, Materials and Methods

DNA and RNA sequences	Information included in the manuscript?	In which section is the information available? (Reagents and Tools Table, Materials and Methods, Figures, Data Availability Section)
**Short novel DNA or RNA including primers, probes**: provide the sequences.	Not Applicable	

Cell materials	Information included in the manuscript?	In which section is the information available? (Reagents and Tools Table, Materials and Methods, Figures, Data Availability Section)
**Cell lines:** Provide species information, strain. Provide accession number in repository **OR** supplier name, catalog number, clone number, and/**OR** RRID.	Yes	Reagents and Tools Table, Materials and Methods
**Primary cultures:** Provide species, strain, sex of origin, genetic modification status.	Not Applicable	
Report if the cell lines were recently **authenticated** (e.g., by STR profiling) and tested for mycoplasma contamination.	Not Applicable	

Experimental animals	Information included in the manuscript?	In which section is the information available? (Reagents and Tools Table, Materials and Methods, Figures, Data Availability Section)
**Laboratory animals or Model organisms:** Provide species, strain, sex, age, genetic modification status. Provide accession number in repository **OR** supplier name, catalog number, clone number, **OR** RRID.	Not Applicable	
**Animal observed in or captured from the field:** Provide species, sex, and age where possible.	Not Applicable	
Please detail **housing and husbandry conditions**.	Not Applicable	

Plants and microbes	Information included in the manuscript?	In which section is the information available? (Reagents and Tools Table, Materials and Methods, Figures, Data Availability Section)
**Plants:** provide species and strain, ecotype and cultivar where relevant, unique accession number if available, and source (including location for collected wild specimens).	Not Applicable	
**Microbes:** provide species and strain, unique accession number if available, and source.	Yes	Reagents and Tools Table

Human research participants	Information included in the manuscript?	In which section is the information available? (Reagents and Tools Table, Materials and Methods, Figures, Data Availability Section)
If collected and within the bounds of privacy constraints report on age, sex and gender or ethnicity for all study participants.	Not Applicable	

Core facilities	Information included in the manuscript?	In which section is the information available? (Reagents and Tools Table, Materials and Methods, Figures, Data Availability Section)
If your work benefited from core facilities, was their service mentioned in the acknowledgments section?	Not Applicable	

Design

Study protocol	Information included in the manuscript?	In which section is the information available? (Reagents and Tools Table, Materials and Methods, Figures, Data Availability Section)
If study protocol has been **pre-registered, provide DOI in the manuscript**. For clinical trials, provide the trial registration number **OR** cite DOI.	Not Applicable	
Report the **clinical trial registration number** (at ClinicalTrials.gov or equivalent), where applicable.	Not Applicable	

Laboratory protocol	Information included in the manuscript?	In which section is the information available? (Reagents and Tools Table, Materials and Methods, Figures, Data Availability Section)
Provide DOI OR other citation details if **external detailed step-by-step protocols** are available.	Not Applicable	

Experimental study design and statistics	Information included in the manuscript?	In which section is the information available? (Reagents and Tools Table, Materials and Methods, Figures, Data Availability Section)
Include a statement about **sample size** estimate even if no statistical methods were used.	Not Applicable	
Were any steps taken to minimize the effects of subjective bias when allocating animals/samples to treatment (e.g. **randomization procedure**)? If yes, have they been described?	Not Applicable	
Include a statement about **blinding** even if no blinding was done.	Not Applicable	
Describe **inclusion/exclusion criteria** if samples or animals were excluded from the analysis. Were the criteria pre-established?<br><br>If sample or data points were omitted from analysis, report if this was due to attrition or intentional exclusion and provide justification.	Not Applicable	
For every figure, are **statistical tests** justified as appropriate? Do the data meet the assumptions of the tests (e.g., normal distribution)? Describe any methods used to assess it. Is there an estimate of variation within each group of data? Is the variance similar between the groups that are being statistically compared?	Yes	Materials and Methods

Sample definition and in-laboratory replication	Information included in the manuscript?	In which section is the information available? (Reagents and Tools Table, Materials and Methods, Figures, Data Availability Section)
In the figure legends: state number of times the experiment was **replicated** in laboratory.	Yes	Figures
In the figure legends: define whether data describe **technical or biological replicates**.	Yes	Figures

Ethics

Ethics	Information included in the manuscript?	In which section is the information available? (Reagents and Tools Table, Materials and Methods, Figures, Data Availability Section)
Studies involving **human participants**: State details of **authority granting ethics approval** (IRB or equivalent committee(s), provide reference number for approval.	Not Applicable	
Studies involving **human participants**: Include a statement confirming that **informed consent** was obtained from all subjects and that the experiments conformed to the principles set out in the WMA Declaration of Helsinki and the Department of Health and Human Services Belmont Report.	Not Applicable	
Studies involving **human participants:** For publication of **patient photos**, include a statement confirming that consent to publish was obtained.	Not Applicable	
Studies involving experimental **animals**: State details of **authority granting ethics approval** (IRB or equivalent committee(s), provide reference number for approval. Include a statement of compliance with ethical regulations.	Not Applicable	
Studies involving **specimen and field samples:** State if relevant **permits** obtained, provide details of authority approving study; if none were required, explain why.	Not Applicable	

Dual Use Research of Concern (DURC)	Information included in the manuscript?	In which section is the information available? (Reagents and Tools Table, Materials and Methods, Figures, Data Availability Section)
Could your study fall under dual use research restrictions? Please check biosecurity documents and list of **select agents and toxins** (CDC): https://www.selectagents.gov/sat/list.htm	Not Applicable	
If you used a select agent, is the security level of the lab appropriate and reported in the manuscript?	Not Applicable	
If a study is subject to dual use research of concern regulations, is the name of the **authority granting approval and reference number** for the regulatory approval provided in the manuscript?	Not Applicable	

Reporting

The MDAR framework recommends adoption of discipline-specific guidelines, established and endorsed through community initiatives. Journals have their own policy about requiring specific guidelines and recommendations to complement MDAR.

Adherence to community standards	Information included in the manuscript?	In which section is the information available? (Reagents and Tools Table, Materials and Methods, Figures, Data Availability Section)
State if relevant guidelines or checklists (e.g., **ICMJE, MIBBI, ARRIVE, PRISMA**) have been followed or provided.	Not Applicable	
For **tumor marker prognostic studies,** we recommend that you follow the **REMARK** reporting guidelines (see link list at top right). See author guidelines, under 'Reporting Guidelines'. Please confirm you have followed these guidelines.	Not Applicable	
For **phase II and III randomized controlled trials**, please refer to the **CONSORT** flow diagram (see link list at top right) and submit the CONSORT checklist (see link list at top right) with your submission. See author guidelines, under 'Reporting Guidelines'. Please confirm you have submitted this list.	Not Applicable	

Data Availability

Data availability	Information included in the manuscript?	In which section is the information available? (Reagents and Tools Table, Materials and Methods, Figures, Data Availability Section)
Have **primary datasets** been deposited according to the journal's guidelines (see 'Data Deposition' section) and the respective accession numbers provided in the Data Availability Section?	Yes	Data Availability Section
Were **human clinical and genomic datasets** deposited in a public access-controlled repository in accordance to ethical obligations to the patients and to the applicable consent agreement?	Not Applicable	
Are **computational models** that are central and integral to a study available without restrictions in a machine-readable form? Were the relevant accession numbers or links provided?	Not Applicable	
If publicly available data were reused, provide the respective **data citations in the reference list**.	Not Applicable	
